# Adaptive designs in clinical trials: a systematic review-part I

**DOI:** 10.1186/s12874-024-02272-9

**Published:** 2024-10-04

**Authors:** Mohamed Ben-Eltriki, Aisha Rafiq, Arun Paul, Devashree Prabhu, Michael O. S. Afolabi, Robert Baslhaw, Christine J Neilson, Michelle Driedger, Salaheddin M Mahmud, Thierry Lacaze-Masmonteil, Susan Marlin, Martin Offringa, Nancy Butcher, Anna Heath, Lauren E Kelly

**Affiliations:** 1 Department of Pharmacology and Therapeutics, Max Rady College of Medicine, University of Manitoba, Winnipeg, MB Canada; 2 George and for Fay Yee Centre Healthcare Innovation, Winnipeg, MB Canada; 3 Cochrane Hypertension Review Group, Therapeutic Initiative, University of British Columbia, Vancouver, BC Canada; 4 Department of Pediatrics and Child Health, University of Manitoba, Winnipeg, Manitoba Canada; 5 Department of Community Health Sciences, University of Manitoba, Winnipeg, MB Canada; 6 Neil John Maclean Health Sciences Library, University of Manitoba, Winnipeg, MB Canada; 7 Department of Pediatrics, Cumming School of Medicine, University of Calgary, Alberta, Canada; 8 Clinical Trials Ontario, Toronto, Ontario Canada; 9 Children’s Hospital Research Institute of Manitoba, Winnipeg, MB Canada; 10 Department of Paediatrics, Management & Evaluation, Institute of Health Policy, University of Toronto, Ontario, Canada; 11 The Hospital for Sick Children, Toronto, Ontario Canada; 12 Department of Psychiatry, University of Toronto, Toronto, Ontario Canada; 13 Division of Biostatistics, Dalla Lana School of Public Health, Child Health Evaluative Sciences, University of Toronto, ScientistToronto, Ontario Canada; 14 Department of Statistical Science, University College London, London, UK; 15 Departments of Pharmacology and Therapeutics, Community Health Sciences, University of Manitoba, 417-753 McDermot Ave, Winnipeg, Manitoba R3E0T6 Canada

**Keywords:** Clinical trials, Adaptive designs, Systematic reviews, Child health, Descriptive analysis, Challenges

## Abstract

**Background:**

Adaptive designs (ADs) are intended to make clinical trials more flexible, offering efficiency and potentially cost-saving benefits. Despite a large number of statistical methods in the literature on different adaptations to trials, the characteristics, advantages and limitations of such designs remain unfamiliar to large parts of the clinical and research community. This systematic review provides an overview of the use of ADs in published clinical trials (Part I). A follow-up (Part II) will compare the application of AD in trials in adult and pediatric studies, to provide real-world examples and recommendations for the child health community.

**Methods:**

Published studies from 2010 to April 2020 were searched in the following databases: MEDLINE (Ovid), Embase (Ovid), and International Pharmaceutical Abstracts (Ovid). Clinical trial protocols, reports, and a secondary analyses using AD were included. We excluded trial registrations and interventions other than drugs or vaccines to align with regulatory guidance. Data from the published literature on study characteristics, types of adaptations, statistical analysis, stopping boundaries, logistical challenges, operational considerations and ethical considerations were extracted and summarized herein.

**Results:**

Out of 23,886 retrieved studies, 317 publications of adaptive trials, 267 (84.2%) trial reports, and 50 (15.8%) study protocols), were included. The most frequent disease was oncology (168/317, 53%). Most trials included only adult participants (265, 83.9%),16 trials (5.4%) were limited to only children and 28 (8.9%) were for both children and adults, 8 trials did not report the ages of the included populations. Some studies reported using more than one adaptation (there were 390 reported adaptations in 317 clinical trial reports). Most trials were early in drug development (phase I, II (276/317, 87%). Dose-finding designs were used in the highest proportion of the included trials (121/317, 38.2 %). Adaptive randomization (53/317, 16.7%), with drop-the-losers (or pick-the-winner) designs specifically reported in 29 trials (9.1%) and seamless phase 2-3 design was reported in 27 trials (8.5%). Continual reassessment methods (60/317, 18.9%) and group sequential design (47/317, 14.8%) were also reported. Approximately two-thirds of trials used frequentist statistical methods (203/309, 64%), while Bayesian methods were reported in 24% (75/309) of included trials.

**Conclusion:**

This review provides a comprehensive report of methodological features in adaptive clinical trials reported between 2010 and 2020. Adaptation details were not uniformly reported, creating limitations in interpretation and generalizability. Nevertheless, implementation of existing reporting guidelines on ADs and the development of novel educational strategies that address the scientific, operational challenges and ethical considerations can help in the clinical trial community to decide on when and how to implement ADs in clinical trials.

**Study protocol registration:**

10.1186/s13063-018-2934-7.

**Supplementary Information:**

The online version contains supplementary material available at 10.1186/s12874-024-02272-9.

## Introduction

Adaptive Designs (ADs) present an alternative to convention, fixed trial designs. ADs are a type of clinical trial design in which carefully planned changes may occur during the study. Specifically, ADs allow for prospectively planned modification(s) to one or more aspects of the study based on accumulating data from the trial [1]. Previous work has suggested that AD makes trials more flexible, and increases the ability to answer research questions by providing the option to shorten trial duration if answers are learned early, or lengthen the trial to ensure the question is meaningfully answered [2, 3]. These potential benefits can be achieved while preserving the integrity and validity of the trial [2] through careful planning before the trial begins (pre-specification) and with proper adjustment for the possible alterations during the trial [2, 4, 5]. All clinical trials should have stopping rules for safety, but ADs often include stopping rules to terminate arms that are not working (futility) which can prevent exposing more people to ineffective or suboptimal treatments and also provide an ethical justification for increasing the application of ADs [1]. ADs have been strongly recommended by the Food and Drug Administration (FDA) [2].

A defining feature of AD trial is that changes are pre-planned (written into the study protocol) and have pre-defined rules, which allow these modifications to roll out during the trial without additional approvals, such as changes to sample size or the number of treatment arms or the allocation ratio of patients to different treatment arms [2], as well as early termination of the trials if an intervention is not safe, or not effective. Additionally, ADs can often provide information about the effectiveness, futility, and safety of interventions earlier than fixed designs, due to an increased number of interim analyses. Earlier identification of ineffective therapies, can cut down on the overall participant burden and cost of a trial [6] and limit exposure to unsafe interventions [7].

Despite several potential advantages over conventional trial designs, the suitability of ADs depends largely on the clinical question being addressed [2]. There are challenges to designing and operating AD clinical trials, including an increased amount of time to design, logistic challenges to preserve trial integrity, ethical considerations and the need to prospectively consider the statistical complexity that each adaptation will bring about.

Although there are many different kinds of adaptations and ADs that have been previously described. Although AD trials share some of the common features (for example that changes are preplanned), the specific trial objectives and research question will determine the acceptability and complexity of adaptation(s) implemented. For instance, adaptive dose-finding trials seek to identify the effective target doses for each patient type by minimizing the dispersion of date around the right dose across different patients [4]. Adaptive randomization designs are a type of AD where pre-specified modifications in treatment allocations vary with the accrued response data (response-adaptive randomization) [5]. These modifications shift the randomization ratio in favour of more participants being allocated to the trial arm with the most promising risk/benefit ratio [6].

Another feature that can be applied in AD trials is group sequential design. These trials incorporate specific stopping rules that specify when to stop trials early for safety, futility (drop-the-loser), or efficacy (pick-the-winner). Seamless designs combine initial safety/efficacy data gathering (phase II) and confirmatory phases (Phase III) into one trial protocol for further investigations in the subsequent trial stages [9]. Seamless designs offer an efficient way to reduce sample sizes for dose optimization and accelerate the development of targeted agents using shared trial infrastructure[10]. A practical advantage of the seamless adaptive trial is that it does not need two separate clinical trial applications, approvals or set-up procedures at study centres, thereby reducing the time taken to evaluate a new intervention[11]. In adaptive enrichment designs, the trial inclusion criteria are modified such that trial participants who have a higher likelihood to benefit from the intervention are increased or “enriched”. However, this may lead to a high uncertainty about the treatment effects in populations who are not “enriched” and may induce statistical bias; adaptive sample size re-estimation designs have been developed address potential biases from adaptive enrichment designs [12]. Sample size re-estimation may be done either in a blinded or unblinded manner based on the criteria of treatment effect-size, conditional power and/or reproducibility probability [2].

Given the limited available resources to conduct clinical trials, and the large number of clinical decisions that are made without population specific evidence, there is an urgent need for trial designs that present efficient and feasible alternatives while meeting ethical, regulatory, and methodological standards [2, 3, 5]. This systematic review seeks to capture the methodological features, study characteristics and reported barriers in real-world examples of trials using ADs. This will help familiarize clinical trialists, ethics boards, regulators and other interested parties with examples of trials that incorporate AD.

## Methods

### Protocol registration

The protocol for this review was published [13] in October 2018 10.1186/s13063-018-2934-7

### Search strategy

We performed a systematic review, reporting according to the Preferred Reporting Items for Systematic Reviews and Meta-Analyses guidelines (PRISMA) [8]. Initially, a health science librarian (CJN) conducted a comprehensive systematic search of the literature on April 10, 2020. The initial search was designed in Ovid Medline and peer-reviewed following PRESS guidelines as outlined in the study protocol [13]. The original strategy was limited to full-text manuscripts on drugs and vaccines in English, French- and Dutch-languages and published between 2010 and 2017. We found a small number (n=16) of randomized controlled trials (RCTs) that specifically reported an “adaptive design” as a keyword. After testing our pre-defined search strategy, we realized that the AD methods are often not specifically reported as such in the keywords, title and abstract of papers. To overcome this, the results of this search were peer-reviewed by a second librarian and the search was further refined. To our knowledge we present a novel systematic review protocol for adaptive trials. This search strategy included was modified from our initial published protocol [13].

The final search included manuscripts published up to April 2020, conducted in the following databases: Ovid MEDLINE(R) and Epub Ahead of Print, In-Process & Other Non-Indexed Citations and Daily <1946 to April 20, 2020>; Embase <1974 to 2020 Week 16>; and International Pharmaceutical Abstracts <1970 to April 2020>. The initial protocol indicated that two additional databases – CENTRAL (Wiley) and MathSciNet – would be used for this review, however, given the large volume of results, these databases were excluded. In addition, the original intent was to limit results to English-, French- and Dutch-language published between 2010 and 2017. The final search was limited to human studies, published in English, French, or Dutch from 2010 to April 2020. Readers are invited to visit the University of Manitoba institutional repository [14] for the complete search histories.

### Selection procedures

Four reviewers (MA, AR, AP, DP) independently reviewed the search results for studies to be included. We screened the titles and abstracts of the 23,886 citations independently, in duplicate. Published protocols, reports, and a secondary analyses that reported using an adaptive method were selected. We included designs with one or more modifications of the trial such as the sample size, the number of treatments, or the allocation ratio to different arms. All diseases and populations (children and adults) were included. Interventions were limited to drugs and vaccines because there are separate regulatory guidance and ethical considerations for device and behavioural interventions in Canada and around the world. Comparator (control groups) were not restricted and we did not include any trial registrations (e.g., ClinicalTrials.gov) as there was not enough data provided on registrations to characterize methodological features.

### Data extraction and syntheses

Four reviewers (MBE, AR, AP, DP) extracted data using standardized data extraction forms in REDCap [15] following the appropriate training and approvals by the University of Manitoba. Discrepancies or disagreements were resolved by rechecking the data, discussion, and consensus between all authors, with adjudication by an arbiter (MBE or LEK) in the event of non-consensus. Data abstracted from included trial details including the key and basic study characteristics of the design, population (age group, disease, location of recruitment), statistical analysis plan, nature of the interventions and control groups, primary and secondary outcomes, planned sample size and sample size methodology, intended analysis, type of adaptation(s), the rationale for adaptation where available, reported challenges, and study limitations that were categorized as regulatory, ethical, logistical, statistical, or other. The strength of the included body of evidence was not evaluated, as the purpose of this study is primarily descriptive.

## Results

### Search results

**Fig. 1:**
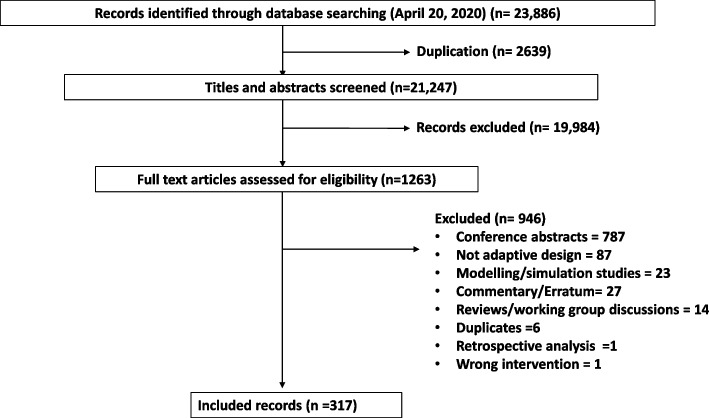
Study Flow diagram, results of literature search

The literature search resulted in 23,886 citations, 2639 removed during deduplication, and 19,984 citations were excluded as they did not meet our inclusion criteria. If there was uncertaintly regarding the application of adaptive designs, full text articles were retrieved. There were 1263 full text articles that underwent a detailed evaluation for inclusion, and a total of 317 trial report were included. See Fig. 1 for the PRISMA study flow diagram [16].

### Description of the included studies

The included 317 publications, were mostly 267/317 (84.2%) clinical trial reports, and there were 50/317 (15.8%) study protocols. There were 390 adaptations reported in 317 studies. Several included trials reported publishing additional information including “appendices and online supplements” 186/317 (58.7%) or “statistical analysis plan” 18/317 (5.7%), and another publication including study protocols 62/317 (19.6%). A large number of these reports were open-access publications (187/317, 59.9%). Most were registered with a clinical trial registry (247/317, 77.9%). Characteristics of included studies are found in Table 1.

**Table 1:** Characteristics of the included studies (*N*=317)

Characteristic	Category (N, %)	N	%
Publication type	Full report	267	84.2
	Study protocol	50	15.8
Additional information published	Appendices/online supplement	183	57.7
	Statistical analysis plan	17	5.4
	Another publication (incl. study protocol)	60	18.9
	Other	30	9.5
	None	74	23.3
Trial registration	Yes	242	76.3
	No	29	9.1
	Unclear	8	2.5
	Not reported	38	12.0
Location of the study participants	Africa	19	6.0
	Asia	54	16.5
	Australia/New Zealand	19	6.0
	Europe	123	39.0
	North America	198	62.9
	South America	15	4.8
	Not reported	6	1.9
Number of centres	Multi-centre	193	61.1
	Single centre	66	20.9
	Not reported	57	18.0
Population	Children only	16	5.4
	Adults only	265	83.9
	Both children and adults	28	8.9
	Unclear	8	2.5
Intervention used	Drug or biologic	315	99.4
	Vaccine	3	0.9
	Surgery	3	0.9
	Radiotherapy	22	6.9
	Medical device	0	0.0
Indication/ Therapeutic area	Oncology	168	53
	Cardiology	6	1.9
	Vascular and hematology	8	2.5
	Virology	16	5.0
	Diabetes	7	2.2
	Others	112	35.3
Duration of intervention	Single dose	33	10.4
	1 – 30 days	99	31.2
	31 – 90 days	53	16.7
	91 – 365 days	109	34.4
	366 – 798 days	18	5.7
	More than 3 years	2	0.6
	Intervention duration not reported	36	11
Control arm	Placebo	84	26.5
	Active drug	58	18.3
	Vaccine	2	0.6
	Surgery	3	0.9
	Radiotherapy	9	2.8
	Medical device	0	0.0
	Historical control	7	2.2
	Standard of care	15	4.7
	No treatment	13	4.1
	No control arm	158	49.8
Study design			
Phase of trial	Phase I	124	39.1
	Phase II	152	47.9
	Phase III	56	17.7
	Phase IV	9	2.8
	Not reported	27	8.5
	Unclear	0	0.0
Description of trial design	Parallel	171	54.3
	Cluster	1	0.3
	Factorial	1	0.3
	Other	124	39.4
	Unclear	1	0.3
	Not reported	17	5.4
Platform trial	Yes	8	2.5
	No	307	96.8
	Unclear	2	0.6
Blinded	Yes	104	32.8
	No	204	64.4
	Unclear	9	2.8
Participants were provided some compensation	Yes	5	1.6
	No	116	36.7
	Unclear	14	4.4
	Not reported	181	57.3
Conflicts of Interest were declared	Yes	208	65.6
	No	85	26.8
	Unspecified	24	7.6
Funding source was declared	Yes	266	83.9
	No	44	13.9
	Unclear	7	2.2
Funder type	Government	95	35.7
	Academic or research institute	63	23.7
	Private	74	27.8
	Industry	131	49.2
	Unclear	4	1.5
	Other	11	4.1

Most studies were conducted in North America 140/317 (44.2%), followed by Europe 56 (17.7%), Asia 25 (7.9%), Australia 20 (6.3%), with the remaining trials conducted in Africa 19 (6.0%) and South America 15 (4.8%). Location was not reported in 6 trials (1.8%). The majority (n=193/317 studies, 61.1%) of included trials were multicentre. Most of the included studies had a relatively short treatment duration of fewer than 30 days (n=132/317, 41.64%), 30 to 90 days (n=53/317, 16.7%) and 90 to 365 days in 109 studies (34.4%). Intervention duration was not reported in 36 studies (11%). The mean duration of follow-up ranges from a single dose one day study to up to 798 days, up to a maximum of 1512 days, with follow-up time ranging from a single day up to 2400 days.

Tables 2 and 3 include a summary of data reported in the adaptive design clinical trials for further description regarding the adaptation designs used. The majority of trials 265 of 317 (83.9%) were limited to adults (over 18 years of age), 16 trials were limited to only children (under 18 years of age), and 28 enrolled both children and adults, 8 trials did not report the age of the participants. The age range across all included trial included from neonates (0-28 days) to geriatric patients (80 years). 169 studies were performed in cancer patients, 17 studies in the therapeutic area of virology, 8 in vascular hematology, 7 studies in diabetes, and 6 in cardiology. Almost all (315/317 = 99.4%) investigated drugs or biologics, three investigated vaccines, and 22 (6.9%) investigated both drugs/biologics and vaccines in combination with radiotherapy or surgery. Of the 317 included studies, 171/317 (53.9%) used parallel groups, and 124 /317 were single-arm (39.1%) studies.

**Table 2:** Summary of the adaptation’s designs* described in included trials (*N*=317)

**Type of adaptation **	*N*	**%**
Adaptive dose‐finding (or dose ranging)	121/317	38.2
Continual reassessment method	60/317	18.9
Adaptive randomization which includes outcome or response‐adaptive	53/317	16.7
Group sequential design	47/317	14.8
Play the winner or drop the loser	29/317	9.1
Seamless phase 2–3 design	27/317	8.5
Pre-trial evaluation	18/317	5.7
• Modelling	4/18	22.2%
• Simulation	13/18	72.2%
• Historical data	1/18	5.6%
Sample size re‐estimation	14/317	4.4
Bayesian logistic‐regression method	6/317	1.9
Biomarker‐adaptive dose‐escalation, 3+3	4/317	1.3
Population enrichment	2/317	0.6
Adaptation not clearly reported	4/317	1.3

*Some studies reported using more than one design. Some ADs fall into more than the category of trial adaptation and were categorized as described in the publication

**Table 3:** Summary of data of adaptive design considerations (*N*=317)

	**N**	**%**
**Were there any unplanned changes to the trial design?**	22/317	7
Examples of unplanned changes:	3	13.6
• sample size	1	4.5
• inclusion criteria	1	4.5
• Randomization	3	13.6
• intervention dose/ administration	1	4.5
• outcomes	1	4.5
• statistical design	1	4.5
• interim analysis	1	4.5
• duration of study	1	4.5
• study sites	1	4.5
**Justification provided for unplanned changes**	21/317	
Reasons provided for unplanned changes were to enroll additional participants, to include additional recruitment sites, to review safety of the drug, to exclude patients who are high risk of GI bleeding, to facilitate enrolment rates or to reduce the time required for participants to stay at the clinic, poor accrual rate and extended funding were the main reasons for addition of new participating sites		95.5
**All adaptation criteria pre-specified**	135/317	42.6
**Special efforts were made to explain adaptiveness to the participants**	3/317	0.9
**Patients or parent/caregivers were consulted during the trial design process**	0	0.0
**Reported a separate adaptation committee (distinct from DSMB)?**	19/317	6
**Who was on the trial adaptation committee?**		
• Researchers/Scientists	10	52.6
• Others/not reported	9	47.4
• Statistician	2	10.5
• Physician/Nurse/Dentist	1	5.3
**Was there a separate trial adaptation committee? Yes**		
**Ref.**	**Who was on the trial adaptation committee and description of these committees**	
[15]	Statistical analysis committee (members NR): The regional coordinating centres forward the data to SAC; and International Steering Committee, the trial is overseen by ITSC, which can add strata, domains and interventions (members NR)	
[16]	Trial Steering Committee (individuals independent of the project and the institutions involved.). The Data Monitoring Committee is using the results of these analyses to advise the Trial Steering Committee on adapting the trial design to either (1) stop prematurely for futility (no prospect of establishing a treatment effect of at least 10%) or (2) stop prematurely if proof beyond a reasonable doubt is established that there is a convincing treatment benefit of at least 10%	
[17]	The institutional review boards (members NR), which for all participating institutions approved the protocol after consultation with the local community and public disclosure.	
[18]	Institutional and National Ethics Committees (members NR). The protocol was approved in Ethiopia, Sudan and UK by the authors of these committees	
[19]	toxicity monitoring committee (members NR)	
[20]	Research Ethics Committee (members NR) Fatal or life-threatening SUSARs will be reported to the Medicines and Healthcare products Regulatory Agency (MHRA) and Research Ethics Committee (REC) within 7 days. The MHRA and REC will be notified immediately if a significant safety issue is identified during the trial	
[21]	Trial Steering Committee, the trial is overseen by a trial, steering committee (TSC) and an independent DSMB to oversee safety and ensure appropriate trial conduct. However, TSC has no role in the implementation of the prespecified adaptive design.	
[22]	Case Assessment and Data Quality and Evaluation Committee (CADQEC), (members NR), which was formed to ensure the integrity and validity of the trial. The CADQEC was entrusted by the sponsor in order to supervise the quality of the data generated at the trial sites before and after unblinding	
[23]	Trial Steering Committee (TSC) (members NR). Sponsor duties are delegated to a trial steering committee comprising the CPI, other investigators and key stakeholders. The DSMC will make recommendations to the trial steering committee via the Coordinating Principal Investigator (CPI)	
[24]	Research and Development Committee of the Michael E. Debakey VA Medical Center (members NR). This trial and all its procedures were approved by the Baylor College of Medicine Institutional Review Board and the Research and Development Committee of the Michael E. Debakey VA Medical Center	
[25]	Ethics Committee (members NR). Ethical approval has been obtained from National and local Ethics Committees in Kenya and Sudan prior to the start of the trial in each Country. A decision for premature termination will be taken in consultation and agreement with the sponsor, investigators and the DSMB. All relevant ethics committees and regulatory authorities will also be informed of the reason for termination.	
[26]	The trial was conducted using a web-based program developed by the Department of Biostatistics and Applied Mathematics at MDACC through which OMCR personnel randomized patients to the 2 arms and updated their current status on an ongoing basis.	
[27]	The TSC (Statistician) will meet at least once annually and will provide overall supervision for the trial and provide advice through its independent Chairperson. The ultimate decision for the continuation of the trial lies with the TSC. The TSC will consist of an independent chairperson (with clinical expertise in HIV), two independent statisticians with expertise in adaptive trial design and medical statistics, a user representative, the investigators, representatives of the research networks, sponsors and principal investigators.	
[28]	An external review board (member NR): The review board recommended a two-group definitive phase 3 design, and the protocol was modified on August 23, 2011, to revise primary and secondary endpoints, sample size and study power, and remove some prespecified stopping rules.	
[29]	A dose- escalation steering committee (member NR) was established to facilitate the trial conduct process	
[30]	The external Statistical Analysis Center (SAC) (member NR) performed all interim data analyses for the DMC, evaluated the decision rules and provided the randomization updates for the adaptive algorithm.	
[1]	Dose-escalation steering committee (member NR) was established to facilitate the trial conduct process	
[31]	The Dose and Frequency Committee (DFC) (Physician, /Nurse/ Dentist, Statistician, Resarcher/Scientist will determine the rules that govern the optimal dose and dose frequency of Proleukin to be given to participants in the next group.	
[32]	Trial Steering Committee (member NR). The study was not originally designed as an adaptive trial however good recruitment and the emergence of data on novel combinations led to trial adaptation. These changes were proposed by the Trial Management Group and approved by the independent Data Monitoring and Ethics Committee and Trial Steering Committee.	

Most trials were in early phase of drug development and were classified as phase I or phase II. Approximately half of the trials (152/317, 47.1%) were phase II trials, mostly in the therapeutic area of oncology, and were aimed at establishing efficacy and choosing doses for the phase III of the trials. 125 studies (38.7%) were phase I trials of new drugs, aimed to assess the safety of treatment across a range of available doses in order to identify the maximum tolerable dose. Only 59/317 (18.6%) of the trials were in phase III, and 10 trials in phase IV (3.1%).

### Summary of adaptative designs used

The most common type of adaptation utilized in these studies was the adaptive dose-finding methodology (121/317, 38.2 %). Some studies utilized more than one adaptation type of adaptive design. Table 2 outlines the adaptations outlined in included studies. Most adaptive dose-finding studies (121/317, 38.2 %), were reported in oncology during the phase I and II stages of the included trials and were evaluating the maximum tolerated dose of the study treatment. Studies using this methodology had relatively small sample sizes studies, ranging from 19 to 889 participants. Continual reassessment methods were commonly used (60/317, 18.9%), aimed to find the maximum tolerated dose from different doses followed by the Bayesian logistic regression approach (6/317,1.9%), and the 3+3 design (4/317, 1.3%).

The second most described method was adaptive randomization design (53/317, 16.7%) which included modification of randomization schedules or treatment, covariate-adaptive randomization, and response-adaptive randomization, in which the participants are increasingly randomized to receive a trial arm which indicates a more promising risk/benefit ratio.

Other designs which were less commonly reported were group sequential multi-stage design (47/317, 14.8%), which aimed to stop the trials early for safety, futility, or efficacy. Similarly, drop-the-losers (pick-the-winner) multi-arm/ multistage designs were used in 29 trials (9.1%) allowing adding additional arms and were mostly used in phase II. These AD designs combine safety and interim treatment selection and confirmatory phases into one trial for further investigations in the subsequent stages.

Adaptive seamless phase II-III designs, which combine a learning stage and a confirmatory stage, was reported in 27 trials (8.5%). Sample size re-estimation design was described in 14 trials (4.4%) where the target sample size was modified based on the observed data in the interim to achieve the desired power. Table 2 outlines the types of adaptions that were reported in included trials.

### Unplanned changes to the trial design

Unplanned changes to the trial design were reported in 22 trials (22/317, 7%) where protocol modifications were made after the recruitment of the first participant. The most common unplanned modification included adding additional recruitment sites. Poor accrual rate and extended funding were the main reasons for the adding new participating sites. Other commonly reported unplanned modification included increase in the sample size, and a change the eligibility criteria or endpoints. Only 6% of studies included other unplanned modifications such as the changes in the statistical design, length of stay of participants, time point of interim analysis, the inclusion of additional doses and modifications to interventions. This modification included either an alternative substitute due to unavailability of the intervention, or a change in the route of administration owing to adverse events. Refer to Additional file 1 for further details on unplanned changes.

### Statistical methods

Approximately 203/317 (64%) studies used frequentist statistical methods and 75/317 (23.7%) used Bayesian statistical methods. Descriptive statistics were used in 17/317 studies (5.4%), which include measures of central tendency such as mean, median, standard deviation, percentage, and correlation. Table 4 includes a detailed summary of the statistical analysis used in the adaptive design clinical trials.

**Table 4:** Summary of the statistical analysis used in the adaptive design clinical trials

Characteristic	N	%
**What was the statistical method used?**		
Bayesian	75	23.7
Frequentist	203	64.0
Descriptive	17	5.4
Unclear	17	5.4
Not reported	5	1.6
**Was there a prespecified plan for statistical handling of missing data?**		
Yes	30	9.5
No	155	4893
Unclear	18	5.7
Not reported	114	36.0
**Was the trial stopped for superiority/non-inferiority?**		
Yes	19	6.0
No	210	66.2
Unclear	21	6.6
Not Applicable	67	21.1
**Was the definition of superiority/non-inferiority prespecified?**		
Yes	53	16.8
No	197	62.3
Unclear	21	6.6
Not Applicable	45	14.2
**Was the trial stopped for futility?**		
Yes	48	15.1
No	209	65.9
Unclear	21	6.6
Not Applicable	39	12.3
**Was the definition of futility prespecified?**		
Yes	90	28.4
No	199	62.8
Unclear	28	8.8
**Were there pre planned interim analysis?**		
Yes	141	44.6
No	84	26.6
Unclear	7	2.2
Not reported	84	26.6
**Were the interim analysis blinded?**		
Yes	13	9.2
No	45	31.9
Unclear	35	24.8
Not reported	48	34
**Was there a prespecified plan for statistical handling of missing data?**		
Yes	30	9.5
No	155	48.9
Unclear	18	5.7
Not reported	114	36.0

### Stopping boundaries

Various stopping boundaries were reported that allowed for stopping a trial prematurely due to safety, futility/efficacy or both based on the results of the interim analysis. Additional files 2 illustrates examples of clinical trials with adaptive designs with further details and complete descriptions of what is reported in these trials. Of the 317 AD studies included in this systematic review, 131 (131/317, 41%) included stopping boundaries to prematurely terminate the clinical trials for futility, efficacy, superiority, non-inferiority, and/or safety. Most of these rules were based on frequentist criteria such as alpha spending functions, O'Brien-Fleming boundary predictive probability, conditional power, critical value criteria (P values and Z boundaries, confidence interval (CI) or standard error (SE), suboptimal response to therapy as compared to another drug placebo and safety). Most of the studies were part of drug development programs for neoplasms utilizing outcomes including progression-free survival, overall survival, response to therapy (both complete and partial response) and drug safety. Futility stopping boundaries, when an intervention was determined to not likely be effective, were more common than superiority/non-inferiority, futility, efficacy and safety-stopping boundaries.

### Logistical challenges

While there are certainly additional trial planning challenges specific to AD, the most common logistical challenges reported in the extant literature within the period of our review were similar to those reported using non-adaptive designs. These include slow participant recruitment, financial issues, difficulty in the identification of outcomes, and severe side effects due to the medication under investigation [3, 17–19]. Compared to non-adaptive designs, the use of ADs adds logistical challenges to ensuring appropriate trial conduct and trial integrity. These challenges include, but are not limited to termination of the medication production, drug supply for multi-arm studies, low infection rates for biological infectious agents, inadequate clinical research and regional infrastructure, and lack of prior clinical trials experience (based on the International Conference on Harmonisation-Good Clinical Practice (ICH-GCP) guidelines), the stringent regulatory standards of western agencies, community resistance, difficulty reaching remote field sites, or inability of the participants to travel long distances, decrease in the quality of life of the participants and vaccine storage issues. Refer to Additional File 3, Summary of the logistical challenges reported in the included trials, for further details regarding these challenges.

## Discussion

Adaptive designs are complex, and this systematic review highlights the methodological features that have been reported in pediatric and adult trials. We have reviewed the literature on trial designs and described how adaptive design methods are used from 2010 to 2020. The most common forms of adaptive design were dose-finding and adaptive randomization designs. Oncology was the most common clinical area observed to use adaptive designs. Frequentist statistical methods were more commonly used than Bayesian methods, and the most common barrier reported to using Bayesian analysis was insufficient knowledge. The logistical and operational concerns reported in this review are mostly not unique to adaptive designs [20–22]. Recruitment challenges reported in conventional and AD trials highlight an urgent need to better understand the dynamics around investigator/trial team and trial-specific factors that can influence participant engagement in the design and operations of clinical trials. Better communication strategies to help convey the different adaptive methods to members of the clinical research community are needed. This should entail engaging in an iterative design built in close communication and collaboration among clinical experts, patient advocates, regulators, pharmaceutical companies, funders and biostatisticians [23].

Many included studies lacked important information on the type of adaptations, including the rationale with respect to the research question. In addition, there were limitations with reporting specifically on how and when data was analyzed (at what stage of the trial). Often in our included studies, it was not clear when the interim analysis was performed and how the sample size re-estimation and adjustment were done. Thus, it was not possible for us to determine if the adapted inclusion criteria were justified, and or if they might have introduced biases into the study. Moreover, it was often unclear who had access to interim results and how adaptive decisions were made. None of the trials reported clearly how they adjusted and accounted for biases introduced by the adaptive study design. This lack of methodological transparency could potentially jeopardize the integrity and uptake of adaptive trials. Thankfully, in 2020 reporting guidelines for reporting adaptive trials were published which should hopefully improve interpretability in future reviews. [24] This review serves as a snapshot of the reporting gaps, and should be re-evaluated in a future study.

Globally, regulators have called for an increase in the use of modern and efficient trial designs. Clinical trials that use ADs are an attractive option because they can potentially increase trial efficiency. However, there is still a lack of knowledge and acceptance among researchers about ADs [19]. Concerns about the appropriate use of ADs in trials revolve around a lack of knowledge and expertise among researchers, a lack of infrastructure support for the planning of AD control trials, complicated statistical analysis methods that are not widely understood, the lack of suitable software to aid both the design and conduct of trials [2], and concerns on how the funders and regulator’s view ADs. All these factors limit the use of ADs in clinical research, thereby denying trialists and patients the appropriation of the range of benefits that AD clinical trials offer. The acceptability of AD, incurring both the potential benefits and additional challenges, depends on the research question[25, 26]. For instance, group sequential designs that repeatedly assess clinical outcomes over multiple interim analysis is effective in trial cost minimization and patient risk reduction. However, the design is poor at gathering information on long-term treatment effects, effects on secondary end points, and may even produce less precise estimates [27]. Similarly, response adaptive randomization designs that produces unequal sample size across treatment and control arms can impact the statistical efficiency of the trial, likely increasing bias in favor of the arm that over-pools patients after a favorable treatment response [25]. Seamless Phase II/III designs can help minimize delays associated with protocol development, and logistical issues, however, it often restricts the flexibility of modifying the confirmatory phase after trial commencement [28].

An understandable critique of ADs is that the adaptive methodological and/or statistical process can introduce operational biases that may be difficult to predict and control, which may inadvertently make the target population to be shifted with regards to location and scale [17]. Although the use of pre-planned statistical analysis plans including extensive simulation and statistical tools such as the Bonferroni-Holm test procedure (based on conditional error rates of individual treatment–control comparisons) can help control and limit these operational biases and the attendant type 1 errors [29, 30], they may raise scientific concerns resulting from unfamiliarity during grant submissions, review and approval. An increase in logistical constraints in safeguarding the trial conduct and integrity as well as the need for specific analytical methods/simulations (that may not be readily accessible as many trial team lack dedicated support from statisticians with expertise in AD) are additional challenges associated with ADs in practice [1].

There are few published reviews of specific ADs in the literature [2–4, 14, 18, 31]. These papers present focused discussions on an overview of ADs, provide a select description of case studies to illustrate their use, or focus on the statistical considerations, statistical solutions, and simulations of ADs. Our review provides the first comprehensive summary of the use of ADs in adult and pediatric trials and provides a snapshot of AD trial reporting. We hope that it will enhance understanding of and familiarity with adaptive methods of clinical trials for the research community as well as increase the confidence of trialists who choose to incorporate them. In a follow-up to this paper (Part II), we will contrast the application of adaptive designs in trials that enrolled adults with those of pediatric populations, with the goal of informing viable educational strategies that can foster trial efficiency and familiarity with AD amongst the clinical research community.

### Limitations of this review

Our search was limited to 2010 to April 2020, based on the FDA guidance [1] on AD in clinical trials that was first issued in 2010, and evaluates its uptake before the adaptive design reporting standards (CONSORT extension) were published in 2020. It is worth mentioning that ADs were used frequently in clinical trials during the COVID-19 pandemic, due to their operational efficiency. However, given the timing of our literature search, COVID-19-related trials are not captured in this review. We also did not attempt to identify trials that are not published or evaluate non-drug/vaccine interventions, which may limit the application of our findings on AD clinical trials to those contexts. Given our focus on trial reports, planned studies that may have encountered barriers to implementing an AD and could not begin at all (e.g., planned trials unable to overcome ethical or regulatory hurdles for the proposed adaptations for approval) would not have been reflected in the published literature. An additional limitation is that publication word counts could have limited how the manuscripts described the rationale for the adaptation-specific challenges.

### Implications for practice

The results of this review suggest that there is a need for rigorous, immersive training in adaptive trial designs for health care providers, researchers, the public and other interested parties who design and implement clinical trials. Proper guidance on planning ADs trials with simulations of possible adaptation scenarios for risk-benefit assessments, supported with clinical input and statistical analysis is necessary to optimize outcomes. In the face of the implementation challenges associated with ADs, developing a framework on how to operationalize them is an important means of overcoming these challenges [17].

### Implications for research

To increase the capacity for adaptive trials, a qualitative study to obtain practical feedback from regulators, research ethics board members, biostatisticians, clinicians, and scientists, as well as representatives from patient groups and the public, on challenges in applying AD in trials to inform recommendations on best practices, is warranted. Given the ethical requirement for monitoring participant safety, an external adaptation committee should be established to continually monitor modifications based on interim data. Guidelines for creating and communicating with adaptation committees are warranted. To increase uptake of AD trials, enforced application of reporting tools and methodological transparency will ensure regulators, trialists and the public can clearly understand the published literature as well as how adaptations are applied and how biases are eliminated (or minimized).

## Conclusions

Clinical trials are needed that can efficiently inform optimal treatment practices. This review provides an overview of the methodological features of adaptive designs that have been reported in clinical trials. Clarity about methodological, operational and ethical features may help increase familiarity and ultimately uptake of trials that incorporate ADs.

## Supplementary Information

Supplementary Material 1

Supplementary Material 2

## Data Availability

No datasets were generated or analysed during the current study.

## Abbreviations

AD: Adaptive design
FDA: Food & Drug Administration
PRISMA: Preferred Reporting Items for Systematic Reviews and Meta-Analyses
MTD: Maximum tolerated dose
ICH-GCP: International Conference on Harmonisation-Good Clinical Practice
CI: Confidence Interval
SE: Standard Error
