# Supplementary material for: Adaptive designs in clinical trials: a systematic review-part I
Source: BMC Med Res Methodol. 2024 Oct 4;24:229. doi: 10.1186/s12874-024-02272-9 (PMC11451232; doi:10.1186/s12874-024-02272-9)
Supplement: Supplementary file 1 — Supplementary Material 1 [file 12874_2024_2272_MOESM1_ESM.docx]

**List of Additional materials**

**Additional File 1:** Summary of the unplanned changes to trial design, all are in adult trials as none of the 16 pediatric trials reported unplanned changes)

**Additional File 2:** Summary of the stopping boundaries and futility definitions reported

**Additional File 3:** Summary of the logistical challenges reported in the included trials

## **Additional File 4:** Summary of amendments (total number of studies that submitted amendments) submitted to the research ethics boards

**Additional materials**

**Additional File 1:** Summary of the unplanned changes to trial design, all are in adult trials as none of the 16 pediatric trials reported unplanned changes)

| **Ref.** | **Study Title** | **Unplanned changes reported to trial design** |
| --- | --- | --- |
| *(34)* | A phase Ib dose-escalation study of everolimus combined with cisplatin and etoposide as first-line therapy in patients with extensive-stage small-cell lung cancer | A protocol amendment was introduced to allow 7.5-mg/day and 40-mg/week doses and mandated prophylactic granulocyte colony-stimulating factor (G-CSF) use from cycle 1 |
| (35) | Idarubicin-loaded beads for chemoembolisation of hepatocellular carcinoma: results of the IDASPHERE phase I trial. | Protocol amendment was done to enrol three additional patients |
| (36) | Randomized, double-blinded, multicenter, phase II study of pemetrexed, carboplatin, and bevacizumab with enzastaurin or placebo in chemo naive patients with stage IIIB/IV non-small cell lung cancer | Second interim analysis was added to review safety of the drug |
| (37) | An adaptive-design dose-ranging study of PD 0348292, an oral factor Xa inhibitor, for thromboprophylaxis after total knee replacement surgery | Protocol was amended to increase in sample size by 200 to allow for adequate sample size in the highest dose group |
| (38) | Phase IB randomized, double-blinded, placebo-controlled, dose-escalation study of polyphenol E in women with hormone receptor-negative breast cancer | Protocol amendment to exclude women with a prior history of GI bleed was made after an episode of grade 3 rectal bleeding occurred in a woman with pre-existing diverticulosis and required hospitalization. |
| (39) | Primaquine to reduce transmission of Plasmodium falciparum malaria in Mali: a single-blind, dose-ranging, adaptive randomised phase 2 trial | Due to low pre-treatment infection rates, eligibility criteria were expanded to include participants aged 5-17 years because this age group is more likely to be infected with microscopically detectable gametocytes. In addition, to maximize the number of mosquitoes for assessment on day 7 for oocyst measurement, the assessment of sporozoites at day 14 was removed. Finally, to facilitate enrolment rates, the time requirement for participants to stay at the clinic was reduced from 26 to 12 hours post treatment |
| (40) | Effectiveness of triclosan-coated PDS Plus versus uncoated PDS II sutures for prevention of surgical site infection after abdominal wall closure: the randomised controlled PROUD trial | Changed from single-centre RCT to multicentre when substantial funding became available from Johnson & Johnson. |
| (41) | A randomized, double blind, placebo-controlled trial of pioglitazone in combination with riluzole in amyotrophic lateral sclerosis | Interim analysis was initially planned after observation of 31 deaths. However, because of the unexpected low number of deaths from start of trial until March 2009, the protocol was amended to perform the interim analysis either after observation of a total of 31 deaths or in June 2009, depending on what condition happens first |
| (42) | Randomized Trial of Central Nervous System-Targeted Antiretrovirals for HIV-Associated Neurocognitive Disorder | Accrual was not on a trajectory to meet the planned goals, and 2 new sites were added in 2010 |
| (43) | Carboplatin and Etoposide With or Without Palifosfamide in Untreated Extensive-Stage Small-Cell Lung Cancer: A Multicenter, Adaptive, Randomized Phase III Study (MATISSE) | Closed enrollment after 188 patients with an amendment due to changes in development plans for palifosfamide. In addition, response assessments and PFS were no longer required as outcomes as part of the amendment. |
| (44) | Safety and immunogenicity of fractional dose intradermal injection of two quadrivalent conjugated meningococcal vaccines | Changed the intervention (vaccine) for booster dose due to unavailability of the required vaccine |
| (45) | BRAINSTORM: A Multi-Institutional Phase 1/2 Study of RRx-001 in Combination With Whole Brain Radiation Therapy for Patients With Brain Metastases | For the first 9 patients, intervention (RRx-001) was given directly intravenously, but owing to localized discomfort and vasodilation on infusion due to localized NO release, the study was amended to change the method of administration. |
| (46) | Carboplatin and Etoposide With or Without Palifosfamide in Untreated Extensive-Stage Small-Cell Lung Cancer: A Multicenter, Adaptive, Randomized Phase III Study (MATISSE) | Closed enrollment after 188 patients with an amendment due to changes in development plans for palifosfamide. In addition, response assessments and PFS were no longer required as outcomes as part of the amendment. |
| (44) | Safety and immunogenicity of fractional dose intradermal injection of two quadrivalent conjugate meningococcal vaccines | Changed the intervention (vaccine) for booster dose due to unavailability of the required vaccine |
| (46) | Adaptive, dose-finding phase 2 trial evaluating the safety and efficacy of ABT-089 in mild to moderate Alzheimer disease | The initially fixed randomization probability for the placebo group was changed from at 0.20 to 0.35 after 12 interim adaptive calculations, in response to unusually higher placebo response rate and recommendation from DSMB. |
| (47) | OV21/PETROC: a randomized Gynecologic Cancer Intergroup phase II study of intraperitoneal versus intravenous chemotherapy following neoadjuvant chemotherapy and optimal debulking surgery in epithelial ovarian cancer. | Revision of the statistical design for the second stage of the trial |
| (48) | Telmisartan to reduce insulin resistance in HIV-positive individuals on combination antiretroviral therapy: the TAILoR dose-ranging Phase II RCT | Amendments were made to include additional recruitment sites and change in exclusion criteria |
| (28) | Addition of sirolimus to standard cyclosporine plus mycophenolate mofetil-based graft-versus-host disease prophylaxis for patients after unrelated non-myeloablative haemopoietic stem cell transplantation: a multicentre, randomised, phase 3 trial | The initially designed 3 group Phase 2 trial in September 2010 was modified to a 2 group definitive phase 3 design in August 2011 after recommendation from an external review board, with revision in the primary and secondary endpoints, sample size and study power, and removing some prespecified stopping rules. 6 patients who were already enrolled in the removed group were not further analyzed. |

**Additional File 2:** Summary of the stopping boundaries and futility definitions reported

| **Ref.** | **What was the indication/s described in the study?** | **Was study eligibility criteria changed throughout the trial?** | **Stopping boundaries (Superiority, futility and efficacy);Stopping boundary safety ;**  **stopping boundaries (suboptimal response)** | **Was the trial stopped for futility?** | **Was the definition of futility prespecified?** | **What was the definition of futility?**  ***What was the definition of superiority/non-inferiority?** |
| --- | --- | --- | --- | --- | --- | --- |
| (49) | Amyotrophic lateral sclerosis | No | Futility: p value ≥ 0·68;  Efficacy: p value of 0·001 | Yes | Yes | p=0·68 |
| (50) | Neoplasm (Glioblastoma) | No | prediction probability: of a positive result gets small. A linear boundary that increases with enrollments from 0 to 0.1 is used to define monthly the cut-off for discontinuing or proceeding with the study of the experimental treatment. | Not Applicable | Yes | Same as SB |
| (51) | Fibromyalgia Syndrome | No | efficacy criteria  study 1: Adjusted mean (SE) change from baseline on the NRS at analysis (LOCF) was -2.09 (0.27) for the placebo group, and the mean (SE) advantage over placebo rangedfrom−0.21 (0.36) for desvenlafaxine 50 mg/d to 0.04(0.35) for desvenlafaxine 400 mg/d  study 2: The study was also for business reasons | Yes | Yes | (Mean [SE] advantage over placebo, -0.21 [0.36] to 0.04 [0.35]) |
| (52) | Neoplasm (metastatic pancreatic adenocarcinoma) | No | safety criteria: If ≤3 of the first 16 patients enrolled to each treatment arm were progression-free at 4 months, that treatment arm would be terminated. | Yes | Yes | If ≤3 of the first 16 patients enrolled to each treatment arm were progression-free at 4 months, that treatment the arm would be terminated |
| (53) | Neoplasms (Non-small-cell lung cancer) | No | Efficacy stopping boundary O’Brien-Fleming the observed p-value is below the adjusted alpha level | Yes | Yes | Unclear |
| (54) | Neoplasm (Relapsed small-cell lung cancer) | Not reported | safety criteria: trial will be stopped if responses were not seen in at least 2 of the first 18 enrolled patients | Yes | Yes | If no responses were seen in the first portion of this Gehan two-stage trial, the trial will not progress to the second stage of the trial and the trial will be appropriately terminated |
| (55) | lumbosacral radiculopathy | Not reported | efficacy criterion (>80% probability that the dose reduces the pain score by =>1.5 points more than the placebo)  futility criterion (<45% probability that the maximum utility dose reduces pain more than the placebo) was met.  80% probability that the maximum utility dose reduces the pain score by >=1.5 points more than the placebo  safety criteria: if 3 dose groups were permanently discontinued because of specified safety criteria | No | Yes | (>45% probability that the maximum utility dose reduces pain more than the placebo) was met. |
| (56) | Blood | No | The observed p-value is below the adjusted alpha level | No | No | - |
| (57) | Asthma | No | clinical FEV1 below stability limit value calculated at randomisation) | No | No | - |
| (58) | Neoplasm (Postpolycythemia vera/post-essential thrombocythemia myelofibrosis) | No | no or one response (complete remissions or major clinical improvement out of first sixteen patients | Yes | Yes | at the evaluation of response at 18 weeks, there were no or one response (complete or major) out of the first sixteen patients, the trial would be terminated for lack of efficacy. |
| (59) | Nervous system/ cerebrovascular diseases | No | If more than 9 out of 15 favourable outcomes were observed at the interim analysis, the study would be halted for efficacy.  safety criteria: Not reported | Yes | Yes | If fewer than 6 favourable outcomes out of 15 were observed at the interim analysis, the study would be halted for futility |
| (60) | Neoplasm (transitional cell carcinoma of the urothelium) | No | In the interim analysis a minimum of 15% response rate in the cabazitaxel group (interim efficacy analysis) | Yes | Yes | In the first preliminary analysis, if one or fewer responses were observed in the cabazitaxel arm, the study should have be stopped (interim futility analysis) |
| (61) | Neoplasms (advanced anaplastic thyroid cancer) | No | one or no confirmed response among the first 14 potential patients | Yes | No | response rate |
| (62) | Neoplasm (malignant gliomas) | No | disease progression, noncompliance safety criteria: toxicity | No | No | - |
| (59) | Hot Flashes in Breast Cancer Survivors | No | if 19 or fewer patients (or if 37 or more patients) preferred gabapentin over venlafaxine, the trial will be stopped Pocock stopping rule | No | No | - |
| (61) | addictive and depressive disorders | No | safety criteria: serious adverse events (SAEs) and adverse events of specific interest (AESI) (eg, terminating the study if >=25% of a dosing cohort experienced a possibly or definitely drug related AESI, such as NSVT) | Yes | Yes | if >=25% of a dosing cohort experienced a possibly or drug related AESI, such as NSVT |
| (64) | Neoplasm (Acute myeloid leukaemia) |  | HR less than 1 | Yes | Yes | HR greater than 1 |
| (65) | Neoplasm (Recurrent glioblastoma) | No | safety criteria: none of first 5 patients enrolled are alive and progression-free at 6 months  efficacy boundary. The study terminated if none of the initial 5 subjects was free of progression and alive at 6 months. | No | No | NA |
| (66) | Neoplasm (metastatic renal cell carcinoma) | No | safety criteria: unacceptable toxicity | No | No | NA |
| (67) | chronic graft-versus-host disease | No | The Z-statistic comparing complete/partial response rates (51% versus 50%, Z=0.11, stopping boundary Z6≤0.9) did not support proceeding to phase III. | Yes | Yes | Z6≤0.9 |
| (36) | Neoplasm (Stage IIIB/IV non-small cell lung cancer) | No | A PFS HR of the enzastaurin arm versus the placebo arm of greater than 0.8857 was the efficacy criterion for futility. Drug safety | Yes | Yes | A PFS HR of the enzastaurin arm versus the placebo arm of greater than 0.8857 was the efficacy criterion for futility |
| (68) | Neoplasm (pancreatic cancer) | No | safety criteria: An added measure of safety was employed such that if there was more than a 0.95 probability that the DLT rate of the first dose exceeded 25% the study would be stopped | No | Yes | An added measure of safety was employed such that if there was more than a 0.95 probability that the DLT rate of the first dose exceeded 25% the study would be stopped |
| (69) | Neoplasm (refractory colorectal cancer) | No | If only one of 30 patients achieved an objective response, the trial would be terminated with the conclusion that there was little evidence to suggest the ORR would reach 15% | Yes | Yes | If only one of 30 patients achieved an objective response, the trial would be terminated with the conclusion that there was little evidence to suggest the ORR would reach 15% |
| (70) | Neoplasm (Malignant neoplasms of rectosigmoid junction, unspecified) | No | Rho family error spending function with ρ=1.5 Progression-free survival will be compared between treatment groups using a log-rank test converted to a z-score and compared with the nominal critical value of 2.372 based on the rho family error spending function corresponding to a one-sided P ≤.0088, allowing the study to be stopped early for efficacy, in which case hepatic progression-free survival will be tested at the same boundary as progression-free survival using a log-rank test converted to a z-score. A second interim analysis is planned at 241 progression-free survival events, where progression-free survival will be compared between treatment groups using a log-rank test converted to a z-score and compared with the nominal critical value of 2.330 based on the rho family error spending function corresponding to a one-sided P ≤.0099, allowing the study to be stopped early for efficacy. If the study is stopped early for progression-free survival at the second interim analysis, hepatic progression-free survival will be tested using the boundary derived based on an incremental alpha of .0057. This boundary will account for the correlation between the z-score for progression-free survival at the first interim analysis and the z-score for hepatic progression-free survival at the second interim analysis, which is determined by the observed number of hepatic progression-free survival events at the first interim analysis and the cumulative number of hepatic progression-free survival events observed at the second interim analysis | No | No | - |
| (71) | Neoplasm (Unresectable, Locally Advanced, or Metastatic Soft Tissue Sarcoma) | No | safety criteria: Cardiotoxicity | Not Applicable | No | - |
| (72) | Neoplasm (acute myeloid leukaemia) | No | The early stopping rules were to terminate treatment within each experimental arm if, compared to the historical experience, that arm's CR rate is unlikely to increase by a mean of 0.15. This rule was applied in each experimental arm after each cohort of five patients, up to a maximum of 15 per arm, was evaluated. The stopping bounds generated by these rules were designed to terminate accrual to an arm if the CR rate was ≤0/5, 1/10, 2/15, 3/20, 3/25, 4/30, 5/35, or 5/40. | Not Applicable | Yes | The trial was to terminate treatment within each experimental arm if, compared to the historical experience, that arm’s CR rate is unlikely to increase by a mean of 0.15. This rule was applied in each experimental arm after each cohort of five patients, up to a maximum of 15 per arm, was evaluated. The stopping bounds generated by these rules were designed to terminate accrual to an arm if the CR rate was ≤0/5, 1/10, 2/15, 3/20, 3/25, 4/30, 5/35, or 5/40. |
| (73) | Neoplasm  (Acute Myeloid Leukemia) | Not reported | safety criteria: the trial to stop early if there are at least 12 patients who received a dosage at the MTD level and there is strong evidence that the lowest dose is too toxic. The trial stopped early if there was a high chance (greater than 72%) that the posterior probability of DLT at the lowest dose was more than 10% greater than the target DLT rate of 20%. | Not Applicable | No | - |
| (74) | Acute Bacterial Skin and Skin Structure Infections | Not reported | The criterion for terminating the phase 2 study and taking the CEM-102 loading dose to the phase 3 study was a predictive probability of phase 3 success of .80. | Yes | Yes | The criterion for terminating the phase 2 study and taking the CEM-102 loading dose to the phase 3 study was a predictive probability of phase 3 success of ..80. |
| (75) | Amyotrophic lateral sclerosis | Unclear | Efficacy stopping used the alpha spending rule approach. That is, a one-sided type I error of 0.025 (equivalent to a two-sided p=0.05 test). Can be allocated during the course the trial)  Stopping for futility could occur if the trial did not reach the accrual targets, or if both Amyotrophic Lateral Sclerosis Functional Rating Scale-Revised (ALSFRS-R) and survival did not show sufficient trends toward efficacy.  If the dropout rate was so high that the power to show a survival benefit was minimal, the primary endpoint of the trial was to change to compare 12-month changes in ALSFRS-R, and participants were given the option to discontinue study medication at 12 months | Yes | Yes | Stopping for futility could occur if the trial did not reach the accrual targets, or if both Amyotrophic Lateral Sclerosis Functional Rating Scale-Revised (ALSFRS-R) and survival did not show sufficient trends toward efficacy. In addition, if the dropout rate was so high that the power to show a survival benefit was minimal, the primary endpoint of the trial was changed to compare 12-month changes in ALSFRS-R, and participants were given the option to discontinue study medication at 12 months. Accrual targets. After allowing 5 months to reach an accrual rate of 25 participants per month, the Data Safety Monitoring Board (DSMB) could stop the study if the accrual futility boundary of ≥60% was not achieved. Efficacy targets. The trial was considered futile if the co-primary endpoints, survival, and ALSFRS-R slopes failed to cross the futility boundary specified by the beta spending function .05*t. |
| (76) | Rectal Cancer (stage II to III rectal adenocarcinoma) | Unclear | Safety criteria: Had a target dose-limiting toxicity (DLT) rate of 25% at the MTD. toxicity. | No | Unclear | - |
| (77) | Mild to moderate Alzheimer's disease | Not reported | safety criteria: microhemorrhage and and hemosiderosis (ARIA-H amyloid-related imaging abnormalities (ARIA) and parenchymal vasogenic edema and sulcal effusions (ARIA-E) | No | Unclear | - |
| (40) | Surgical site infection after abdominal wall closure | Not reported | If superiority of PDS Plus if the one-sided p value fell below 0·0102 After the second stage, superiority of PDS Plus would be shown if the product of the one-sided p values from the two stages fell below 0·003  futility if the one-sided p value was above 0·5 | No | Yes | At first interim analysis, the trial would stop for futility if the one-sided p value was above 0·5.  The trial would stop with demonstration of superiority of PDS Plus if the one-sided p value fell below 0·0102 at first interim analysis. Otherwise, the trial would continue to a second stage. After the second stage, superiority of PDS Plus would be shown if the product of the one-sided p values from the two stages fell below 0·0038 |
| (78) | Active Ileocolonic Crohn's disease | Not reported | - | No | No | Non-inferiority test with a one-sided significance level of α = 0.025 and a non-inferiority margin of −15% was used, based on the inverse normal method of combining the p-values of the shifted asymptotic χ2 test for comparing two rates and maximum likelihood estimation for the unknown parameters. The confidence interval (CI) limit of 15% was selected based on an expected remission rate of 55% in both the budesonide 9 mg OD group and the 3 mg TID group, well above the reported placebo remission rate of 25% 25,26 assuring a clinically relevant effect superior to a putative placebo and no clinically relevant inferiority to the control group. |
| (79) | Visceral leishmaniasis in HIV co-infected patients | Not reported | Possible stopping after every 10 patients, based on acceptable or unacceptable efficacy values set in the protocol. On crossing the upper boundary, the trial should be stopped for promise (efficacy above 75%), and on crossing the lower boundary it should be stopped for lack of promise (efficacy below 90%). | Unclear | Unclear | - |
| (80) | Ebola virus disease | No | Depending on the expected occurrence of success, failure or insufficient evidence | Not Applicable | No | - |
| (42) | HIV-Associated Neurocognitive Disorder | No | The trial would be stopped for efficacy if the 2-sided P value corresponding to the effect of treatment on GDS change score was ≤.00305 (beneficial or hazardous effect); (2) the trial would be stopped for futility if the conditional power crossed the 40% futility boundary, selected prior to the analysis. | No | Yes | The trial would be stopped for futility if the conditional power crossed the 40% futility boundary, selected prior to the analysis |
| (81) | Higher-Risk Myelodysplastic Syndrome | Not reported | Assignment to a treatment was stopped if the probability that patients were assigned to this arm was <=5% or if there was an indication that a response rate of >=20% was not achieved. | No | No | A treatment would only be selected as superior if there was sufficient evidence of superiority, whereas otherwise the trial would accrue up to a total of 60 patients and would be regarded as inconclusive. |
| (82) | Migraine | No | at least five blocks of subjects had been treated at this dose, and for at least four blocks the decision rule called for a dose decrease. Alternatively, the dose selection process could have been terminated, without the selection of an effective dose, if five consecutive blocks of subjects had been treated at the top dose with the escalation rules calling for a dose increase each time. | Yes | Yes | - |
| (83) | Chronic Diabetic Foot Ulcers | No | The Pocock stopping boundary method requires a more stringent P value threshold (P, 0.022) at each of the three analyses points (after one-third (73), two thirds (146), and finally all (220) enrolled patients) to achieve an overall probability of P, 0.05 at the final evaluation. | No | N0 | - |
| (84) | Mild-to-moderate Alzheimer's dementia | No | - | Unclear | Yes | The study could stop early for futility if the predictive probability of phase 3 success for the most likely EDmax was less than 0.2, or the posterior probability that the mean improvement in the ADAS-Cog of the most likely EDmax was better than donepezil was less than 0.1.  Part 1 of the study could stop early for success if the predictive probability of phase 3 success for the most likely EDmax was at least 0.9 and the posterior probability that the mean improvement in the ADAS-Cog of the most likely EDmax was better than donepezil by at least 0.85. The study could stop early for futility if the predictive probability of phase 3 success for the most likely EDmax was less than 0.2, or the posterior probability that the mean improvement in the ADAS-Cog of the most likely EDmax was better than donepezil was less than 0.1. |
| (85) | Type 2 diabetes mellitus | Not reported | the trial is stopped for futility, if new patients continue to randomize, the treatment allocation probabilities will continue to be updated every 2 weeks and, concomitantly, the algorithm will assess whether the decision rules have been met. Stage 1 will continue until sufficient data have accumulated to make a decision or until 400 patients have enrolled. If the algorithm cannot make a decision after 400 patients, the trial will terminate  If 2 doses of dula are selected, the high dose should be the dose estimated to have the maximum utility, and the low dose should be the lowest dose estimated to have meaningful clinical benefit based on the CUI.14 The low dose cannot be adjacent to the high dose; it must be at least one-half the dose of the high dose as long as efficacy is maintained. If none of the doses fulfill the prespecified decision criteria, the algorithm will terminate the trial for futility.  efficacy (HbA1c) measures by HbA1c change from baseline using a noninferiority margin of 0.25%.  safety criteria: if the patient develops severe and persistent hyperglycemia, acute pancreatitis,  or hepatic or renal impairment, becomes pregnant, or  requires chronic insulin therapy | Not applicable  It’s a protocol | No | Not reported |
| (86) | Progressive primary or recurrent glioblastoma multiforme | Not reported | safety criteria: A progress to the 2nd study group will only take place if there is no objection of the Competent Authority regarding unjustifiable adverse reactions occurred previously | Not applicable | No | - |
| (87) | Unilateral total hip arthroplasty | Not reported | O’ Brien and Fleming. The null hypothesis had to be tested at each interim analysis and the clinical study could be stopped if the one-sided tests yielded p-values lower than respective boundary p-values of each stage  . Statistical significance was set at one-sided a ¼ 0.025 meaning that only an effect in favour of Neodolpasse Infusion Solution was to be interpreted. For a one-sided a ¼ 0.025 and the information rates for each stage, the resulting boundary p-values were p1 ¼ 0.0003, p2 ¼ 0.0071, and p3 ¼ 0.0225  with corresponding critical values of 3.473, 2.454, and  2.004, respectively.  Under these conditions, assuming a mean difference between the treatments of 10 mg morphine in  favour of the Neodolpasse Infusion Solution, with a  common standard deviation of 20 m.  safety criteria: hepatic or renal impairment, becomes pregnant, or or hepatic or renal impairment, becomes pregnant, or requires chronic insulin therapy | No | No | - |
| (88) | Patients with previously untreated ovarian, fallopian tube or primary peritoneal carcinoma | Not reported | 2 stage design: The first stage required 20 patients. If four or fewer patients had adverse events, then the study closed early, and the regimen was deemed feasible. If eight or more patients experienced adverse events, then the study closed, and the regimen was declared not feasible. If 5-7 patients experienced adverse events, the study reopened to a second stage, targeting a cumulative accrual of 40 patients. If 11 or fewer patients had an adverse event, then the regimen was declared feasible. Otherwise, the regimen was declared not feasible.  safety criteria require chronic insulin therapy. | No | Yes | If eight or more patients experienced adverse events, then the study closed and the regimen was declared not feasible. If 5-7 patients experienced adverse events, the study reopened to a second stage, targeting a cumulative accrual of 40 patients. If 11 or fewer patients had an adverse event, then the regimen was declared feasible. Otherwise, the regimen was declared not feasible. |
| (89) | Advanced ovarian cancer and other solid malignancies | Not reported | Trial is stopped, if probability of either of the undesirable outcomes (safety and activity) is strong enough  Stopped for sufficient evidence of final MTD estimation | No | No | - |
| (90) | Small Cell Lung Cancer | Not reported | undesirable outcomes | Unclear | Unclear | - |
| (91) | femoral nerve block | No | discontinuation criteria when the estimated probability of response was either too low or too high for all dose levels; or (iii) when a suitable estimate of the ED95 was obtained, based on the predictive gains (mean and maximum) of the inclusion of further patients on the response probability and on the width of its credibility interval lower than 5%.  Trial is stopped, if probability of either of the undesirable outcomes (safety and activity) is strong enough | No | No | - |
| (92) | Sepsis | Not reported | if the trial were to enroll all 2000 subjects and the predictive probability is less than 10%, the trial will be stopped for futility . If the predictive probability of success on VVFDs for currently enrolled subjects exceeds 95% but the predictive probability of success on mortality is less than 10% should the trial continue to enroll 2000 subjects, the trial will stop for expected success on VVFDs alone since detecting any mortality benefit is likely out of reach.  VVFDs represent a combination of treatment effect on mortality (all deaths are recorded as zero VVFDs) as well as treatment effect on vasopressor and ventilator support dependence in survivors. | Not applicable | Yes | In early interim analysis (at N<500), based on recommendations from DSMB, it can be stopped 2. In late interim analysis (≥ 500) , if the predict probability of success on VVFD (PP vvfd ) is <0.10 it can be stopped for futility |
| (93) | Acute Ischemic Stroke | No | If the selected tenecteplase dose after Phase 2B had >=2 symptomatic ICHs than rtPA at the interim analysis, then the research would stop. | No | Yes | If the proportion of good outcomes for tenecteplase was significantly less than the proportion of good outcomes with rtPA at the nominal 2-tailed 0.001 level in either scenario, then further study of tenecteplase would be declared futile.  If the selected dose of tenecteplase showed a lower symptomatic ICH rate than rtPA, defined as at least 2 fewer symptomatic ICHs, we would declare it promising if the observed proportion of patients with poor 3-month outcome was less than or equal to that of rtPA (Scenario 1). In Scenario 2, if the rate of symptomatic ICH within 24 hours for tenecteplase was effectively the same (ie, +/-1) as that for rtPA, then the proportion of poor outcomes on the 3-month Rankin Scale would have needed to be at least 8 percentage points lower than that of patients with rtPA for further study of tenecteplase to be declared promising. |
| (94) | Acute mania | No |  |  |  |  |
| (95) | Obstructive sleep apnea | No | Our adaptive dose-finding study allowed for early termination due to futility. When approximately 80 patients had the opportunity to complete (40% of total enrollment), the estimated difference of MK-0249 minus placebo was less than 1 min. This number represents approximately 20% conditional power. | Yes | No | Our adaptive dose-finding study allowed for early termination due to futility. When approximately 80 patients had the opportunity to complete (40% of total enrollment), if the estimated difference of MK-0249 minus placebo was less than 1 min. This number represents approximately 20% conditional power. |
| (96) | Non-Hodgkin's lymphomas | No |  | Yes | No | Stop the study as soon as there was a 0.90 probability that one treatment arm had a higher success rate than the other treatment arm |
| (97) | Major Depressive Disorder | No | The study was to be stopped for lack of sufficient benefit (futility) if there was less than a 5% probability that the true treatment difference in the Bech score was greater than 1.6 | Yes | No | The study was to be stopped for lack of sufficient benefit (futility) if there was less than a 5% probability that the true treatment difference in the Bech score was greater than 1.6 |
| (98) | Sepsis-Associated Acute Kidney Injury | Not reported | If none of the 3 recAP doses in Part 1 show evidence of efficacy (i.e., all 3 groups have 1-sided, unadjusted p-value greater than 0.8), then the study will be terminated | No | Yes | At the futility analysis, each recAP dose would only be deemed as having shown some evidence of efficacy if the one-sided, unadjusted p-value for its comparison with placebo is less than 0.8. If no efficacy, further discussion with sponsor and Steering Committee whether the trial should be terminated for futility. |
| (99) | Advanced malignancies/ advanced solid tumours | Not reported | safety | Unclear | Unclear | - |
| (43) | Extensive-Stage Small-Cell Lung Cancer | No | Early stopping for efficacy or futility using O'Brien-Fleming boundary at 0.5 | No | Yes | O’Brien-Fleming boundary at 0.5 for early efficacy or futility |
| (100) | Disorders of the adrenal glands or adrenal hormone system | Unclear | If the probability that progressive disease exceeded 20%  If drug discontinuation exceeded 10%, the trial would be stopped early | Unclear | Unclear | - |
| (101) | Neoplasms | No | If no clinical response was observed in the first six patients, the trial would be stopped by reason of futility, and if five or more clinical responses were observed, it would be stopped for reasons of efficacy. | No | Yes | If no clinical response (defined by a decrease in tumor size by >= 30%) was observed in the first six patients, the trial would be stopped by reason of futility |
| (102) | Neoplasms | No | For any given dose, the escalation stopped if the rate of toxicity appeared to be higher than approximately 30%, and the lower doses were evaluated and/or re-evaluated | Yes | Unclear | NA , it’s a dose escalation study |
| (20) | Malignant neoplasm | No | the trial is stopped early due to unacceptable DLT levels at the lowest dose. | No | No | - |
| (46) | Disorders with neurocognitive impairment as a major feature | Unclear | The study would be stopped for success if there was Z80% probability that a minimum effective dose (MED) had been identified | Yes | Yes | the study would be stopped for futility if there was Z95% probability that no ABT-089 dose group would achieve Z=1.38-point improvement over placebo on the ADAS-Cog total score. The cut-off value for futility was chosen to be less than the cut-off value for success to support the design intent that is to allow a protective zone for potential viable treatment effect. The value of 1.38 was determined because it provided strong operating characteristics for futility stopping as shown by trial simulations. |
| (21) | Septic shock | Unclear | If none of the arms has a predictive probability of at least 25% then the trial is terminated for futility and interpreted as a standalone phase 2b trial.  The study would be stopped for success if there was Z80% probability that a MED had been identified; | Unclear | Yes | If Part 1 of the trial proceeds to the maximum sample size of800 treated patients and no finding of futility or decision to transition to Part 2 has been made, then a decision to stop for futility or to progress to Part 2 is made, using the following rule: if the predictive probability of trial success is at least 25% for the active arm with the highest predictive probability of trial success, then that arm is selected and Part 2 is begun. If none of the arms has a predictive probability of at least 25% then the trial is terminated for futility and interpreted as a standalone phase 2b trial.  The trial design software calculates the predictive probability that the trial will result in statistically significant superiority under the assumption that each of the active arms is immediately carried forward into Part 2. |
| (103) | Neoplasms of haematopoietic or lymphoid tissues | Unclear | <2% probability of both CR rate at least 40% | Unclear | Unclear | - |
| (104) | Prostate Cancer | No | The trial may be stopped at the completion of the Phase II if there are toxicity concerns, no drug activity (i.e., Futility analysis), or a significant finding related to drug activity. | No | Yes | The purpose of the futility analysis is to examine at an early point in the trial (i.e., after Phase II) evidence of no efficacy to determine whether to continue or stop the trial early. This is very important for preventing future patients from undergoing unnecessary procedures and therefore unnecessary risks when there is little chance of the treatment benefit and was initially suggested by the FDA. |
| (105) | Malignant neoplasms | Not reported | If less than 57 patients demonstrated responses in the first 28 evaluable patients, the regimen was terminated early and deemed ineffective. If less than 8 patients showed responses in the first 28 patients, 11 additional patients were treated for a total of 39. If less than 15 of the 39 patients showed responses, the regimen was not recommended for further study. | No | No | If less than 57 patients demonstrated responses in the first 28 evaluable patients, the regimen was terminated early and deemed ineffective. If less than 8 patients showed responses in the first 28 patients, 11 additional patients were treated for a total of 39. If less than 15 of the 39 patients showed responses, the regimen was not recommended for further study. |
| (106) | ischemic stroke | No | safety criteria: Dose limiting toxicity | No | No | - |
| (107) | Certain lower respiratory tract diseases | Not reported | The probability of a dose being futile was calculated. Doses could be stopped for futility if there was <10% chance of there being ≥75 ml improvement over placebo in FEV1 change from baseline | Yes | Yes | The probability of a dose being futile was calculated. Doses could be stopped for futility if there was <10% chance of there being ≥75 ml improvement over placebo in FEV1 change from baseline |
| (108) | XN487 Human immunodeficiency virus | Not reported | safety criteria: The % differences in the safety criteria are chosen so that the chance of seeing a safety difference that warrants stopping a dose is less than 1 in 40 (<2.5%) assuming both GSK744 LA + TMC278 LA dosing regimens have the same ISR safety profile, i.e. chance of false positive is controlled at <2.5% | No | Yes | The proposed futility analysis has 91% chance to discontinue the IM treatment arm at the interim analysis if the true response rates are 92% and 72% for the oral arm and IM arm, respectively. The power to discontinuing the IM treatment arm is 46% if the true response rates are 92% and 82% for the oral arm and IM arm, respectively. The chance of discontinue the IM treatment in error is 2% if the true response rates are 92% for both the oral arm and IM arm. |
| (109) | advanced breast cancer | No | 50% improvement in median PFS with buparlisib versus placebo, corresponding to hazard ratio  (HR) 0.67 in the full and/or PI3K pathway-activated population. | Yes | Yes | meeting protocol-specified criteria for futility in both the full and PI3K pathway-activated population (with predictive powers of 1.8% and 5.2%, both lower than the predefined 35% threshold); phase III was not initiated. |
| (109) | acute pancreatitis | No | The expected number of patients needed to satisfy the stopping criteria, defined as an absolute 10 % benefit compared to our historical controls, was 20-50. | Yes | No | If any of the groups are significantly (p < 0.0294) less effective than the others and it is already visible that there is no hope for ascertaining a significant difference between the other two groups, the study will be stopped.    If any of the groups are significantly (p < 0.0294) more effective than the others, the study will be stopped. |
| (110) | Abnormality of tonus and reflex | Yes | Early success rule, stop for success if the Bayesian probability for any dose satisfies: P(6-month fall rate < 0.15)=0.80 | No | Yes | Early futility rule, stop for futility if the Bayesian probability for all doses satisfies: P (6-month fall rate > 0.20)=0.80 |
| (111) | carcinosarcoma of the uterus | No | If more than three out of 22-24, or more than four out of 25-29 patients had a documented complete or partial response accrual to the second stage was to be initiated. Otherwise, the study was to be stopped and the treatment regimen classified as uninteresting. | No | No | - |
| (112) | Heart failure | No | Early success rule, stop for success if the Bayesian probability for any dose satisfies: P (6- month  fall rate<0.15) =0.80; and 2) Early futility rule, stop for futility if the Bayesian probability for  all doses satisfies: P(6-month fall rate>0.20)=0.80. E | No | No | Study to be stopped: p(l) < .0038 Statistically significant superiority or inferiority of the PCT-guided study arm over the standard of care arm has been established |
| (113) | malignant neoplasms | No | At the interim efficacy look when at least 75% of the total expected events have accrued, the DMC will consider stopping the study early for efficacy if the lower bound of the specified CIs on VEHZ is > 25%. | Yes | Yes | V212-011 is an adaptively designed study which included a planned interim analysis for futility when 50% of the targeted cases of HZ had accrued in each population. |
| (114) | Sleep-wake disorders | No | at any IA, randomization could be stopped for an early signal of success if the Bayesian analysis indicated there was at least 1 dose with at least an 85% probability of having a utility function > 1, and if that dose did not meet the operational definition of unacceptable safety at days 15 and 16. If randomization was not stopped early, success at study completion was defined similarly, except that the probability of the utility function > 1 was only required to be at least 80%. | No | Yes | Adaptations as well as decisions regarding success and futility were based on the maximum utility dose, defined as the dose with highest mean utility |
| (115) | Squamous cell neoplasms, malignant | No | ORR of >20% based on calculation of the Bayesian posterior probability, and a 90% threshold for such a probability was adopted as a stopping rule | No | No | - |
| (116) | Malignant neoplasms of breast | No |  | No | No | The probability of demonstrating the primary non-inferiority hypothesis was assessed by Bayesian analysis of data from N = 120 patients. The resulting low probability (<1%) led to recommended stoppage of the trial |
| (24) | treatment-resistant depression | No | The study Stopping Rule is when the response rate of the best performing condition is large enough that the posterior probability is > 0.975 that it is better than the next best condition. | No | Yes | The study Stopping Rule is when the response rate of the best performing condition is large enough that the posterior probability is > 0.975 that it is better than the next best condition. Conversely, using similarly constructed a priori evidence rules, suspension of accrual will result for conditions demonstrating such poor performance that continuation of the condition is futile.  Based on pre-specified rules, if any condition demonstrates overwhelming evidence of superiority the trial will be stopped for utility |
| (117) | Chronic Lymphocytic Leukaemia (CLL) | No | Simon's two-stage optimal design (12] will be used to set stopping bounds at each stage of the analysis. A negativity rate above 35% would deem the therapy active, and a success rate below 15% would deem the therapy inactive. If hypersensitivity to obinutuzumab occurs (typically occurring after previous exposure to obinutuzumab), the infusion should be stopped, and treatment permanently discontinued. | Not Applicable | No | - |
| (118) | malignant neoplasms | No | a stopping rule defined as either six patients (two cohorts) treated at a dose level in which the CRM recommends the same dose levels again or if the prespecified maximum number of subjects (e.g. 36) is reached. | No | No | - |
| (119) | Graft-versus-host disease | No | After 30 patients had been evaluated, we calculated the predictive probability that pk could ever be judged to be greater than p0, and we dropped treatment arm k if this predictive probability was < 0.05. The study would stop if all pentostatin treatment arms were dropped by this criterion, and pentostatin would have been declared ineffective at any of the doses studied. | No | No | - |
| (120) | Histopathology | No | If two or fewer patients showed evidence of response, the study was to be stopped. (It means less than 20% improvement in  response rate) | Yes | No | - |
| (121) | Acute kidney failure | No | If endogenous creatinine clearance ≥20 mL/min, RRT should be discontinued | Not applicable | No | The study may be terminated for futility if none of the three recAP doses in Part 1 show evidence of efficacy for the primary end point. Each recAP dose will only be deemed futile if the one-sided, unadjusted p value for its comparison with placebo is <0.8. If all of the doses in Part 1 fulfil the futility criterion, then the DMC will recommend further discussion with the sponsor and Steering Committee to determine whether the trial should be terminated for futility. |
| (47) | Epithelial ovarian cancer | No | An i.p. arm would be considered as futile to continue if its PD9 was 5% or greater than that of the i.v. arm, which had an expected PD9 of 40%e  safety criteria: Safety SB >=29 patients failed to complete that i.p. treatment due to toxicity | No | Yes | An i.p. arm would be considered as futile to continue if its PD9 was 5% or greater than that of the i.v. arm, which had an expected PD9 of 40%. If neither i.p. arm met criteria for futility, the arm with lower PD9 would be selected for the second stage unless >=29 patients failed to complete that i.p. treatment due to toxicity |
| (26) | Brain Cancer (glioblastoma) | Not reported |  | No | No | The trial would stop early and a treatment arm “winner" if the posterior probability of the median PFS of that treatment arm being larger than that of the other arm exceeded 0.995. |
| (122) | SCLC progressing after first-line platinum-etoposide chemotherapy. | Unclear | O'Brien-Fleming stopping rules was used. The null hypothesis was rejected if more than 12 of 45 patients were responders. | No | Unclear | The null hypothesis was rejected if more than 12 of 45 patients were responders. |
| (48) | human immunodeficiency virus (HIV) infection | Yes | If any active dose shows no improvement over control (i.e. has an increase in HOMA-IR), that active dose will be dropped from the second stage. 3. If all three active doses do not show no improvement over control (i.e. has an increase in HOMA-IR), then the study will be stopped and no significant improvement over control will be claimed for any of the active doses. | Yes | Yes | For outcome 1: one dose substantially more effective than control...Study stopped: dose recommended for Phase III Outcome 2: all doses less effective than control...Study stopped Outcome 3: one or more doses are more effective than control, but do not meet outcome 1 criteria.... Study continues with effective dose(s) |
| (123) | human immunodeficiency virus (HIV) | Unclear | if no dose showed sufficient promise at the interim analysis, the study would be stopped altogether. | No | Yes | there were 3 different possibilities for stage II: (1) if 1 active-dose group was substantially more effective than the control, then the study would have been immediately stopped and the corresponding dose would be taken directly into phase 3; (2) if any active-dose groups showed insufficient promise at the interim analysis, they would be dropped and the study continued with the remaining doses and control for a further 24 weeks; or (3) if no dose showed sufficient promise at the interim analysis, the study would be stopped al- together. |
| (27) | HIV-positive | No | If any active dose shows no improvement over control (i.e. has an increase in HOMA-IR), that active dose will be dropped from the second stage. 3. If all three active doses do not show no improvement over control (i.e. has an increase in HOMA-IR), then the study will be stopped and no significant improvement over control will be claimed for any of the active doses. | Not Applicable | Unclear | - |
| (124) | HER2-positive breast cancer. | Yes |  | No | No | The trial was designed to detect an increase in the rate of pCR in the 24- week arm from 27% observed at first At the end of the trial, if 20 or more subjects responded, and the pCR rate with extended therapy was numerically superior to the control (12 week) pCR rate, we could conclude in favor of extended therapy. |
| (125) | Breast Cancer | Not reported | at least 6 patients had been treated at a dose predicted to be the MTD; or c) all doses evaluated appeared too toxic and the MTD could not be determined.  safety criteria: all doses evaluated appeared too toxic and the MTD could not be determined. | No | No | - |
| (126) | hyponatremia in cancer patients | Not reported | If the probability that the correction rate, for example, in the tolvaptan arm, was greater than the rate in the control arm, given data that are > 0.975, then the trial would stop, and the tolvaptan arm would be selected as superior. Similarly, a futility rule, if the data favored placebo, also was in place. Once the efficacy data on first 30 evaluable patients became available, the adaptive phase of the study would start, in which more patients would be assigned to the arm that exhibited a higher rate of meeting the primary end- point of serum sodium correction by day 14  For example, simulations revealed that, if the true serum sodium correction rate was 25% in the control arm and 65% in the tolvaptan arm, then the power to detect the superiority of tolvaptan would be 93% if all 120 planned patients were treated (see Supporting Table 1). If the probability that the correction rate, for example, in the tolvaptan arm, was greater than the rate in the control arm, given data that are > 0.975, then the trial would stop, and the tolvaptan arm would be selected as superior. Similarly, a futility rule, if the data favored placebo, also was in place. Once the efficacy data on first 30 evaluable patients became available, the adaptive phase of the study would start, in which more patients would be assigned to the arm that exhibited a higher rate of meeting the primary end- point of serum sodium correction by day 1 | Yes | Yes | - |
| (127) | agitation among patients with schizophrenia or bipolar I disorder | Not reported | Approximately 360 patients will be recruited with an interim analysis conducted once 180 patients have completed the study to decide whether to stop for futility or continue with or without an increase in the sample size up to additional 288 patients. An interim analysis will be performed on the phase 1 data to determine whether to continue into phase 2 or to stop the trial due to futility or unfeasibility. The final sample size of the PLACID study will be re-calculated based on the interim analysis results. If the interim analysis suggests that more than 30% additional patients are required at 0.025 one-tailed alpha level and approximately 90% power, it will be considered if it is feasible to include >30% additional patients. Since there will be neither stopping for over- whelming efficacy, nor is there a situation where the sample size will decrease based on the interim analysis results, the alpha level will be preserved. | Not applicable | Yes | A total sample size of 180 in each group will have approximately 90% power to demonstrate superiority of in- haled loxapine versus IM aripiprazole for the primary efficacy endpoint at a one-sided significance level of 0.025. If the interim analysis suggests that more than 30% additional patients are required at 0.025 one-tailed alpha level and approximately 90% power, it will be considered if it is feasible to include >30% additional patients. Since there will be neither stopping for over- whelming efficacy, nor is there a situation where the sample size will decrease based on the interim analysis results, the alpha level will be preserved |
| (28) | advanced haematological malignancies320 | Yes | The initial study was designed in September 2010, as a phase 2 trial with three study groups: a standard group with mycophenolate mofetil and cyclosporine, a group with sirolimus added to mycophenolate mofetil and cyclosporine, and a group with cyclosporine and sirolimus. However, an external review board recommended a two-group definitive phase 3 design, and the protocol was modified on August 23, 2011, to revise primary and secondary endpoints, sample size and study power, and remove some prespecified stopping rules. Before this change was implemented, six patients had been enrolled in the cyclosporine and sirolimus group. These patients have not been included in the analyses presented. | Yes | Yes | the study was closed prematurely recommendation of the Data and Safety Monitoring Board on July 27, 2016, after 168 patients received the allocated intervention, based on the results of a prespecified interim analysis for futility. The interim futility analysis was designed to stop the trial if the estimated power to show a significant difference between groups for the primary endpoint was less than 33%, given the results at the time and assuming a 15% true difference. Results were analysed on July 1, 2018. Analyses were done per protocol; all randomly assigned patients who received conditioning and HSCT were included in the efficacy and safety analyses.  The interim futility analysis was designed to stop the trial if the estimated power to show a significant difference between groups for the primary endpoint was less than 33%, given the results at the time and assuming a 15% true difference. Results were analysed on July 1, 2018. |
| (128) | persistent/recurrent cervical cancer | No | An optimal flexible 2-stage design by Chen and Ng was chosen for this study to evaluate the null hypothesis [17]. Stage I targeted 12 eligible and evaluable patients with an accrual range from 8 and 15; if 1 or more out of the 12 patients had objective tumor response, the study would advance to the second stage. Stage II targeted an overall 22 eligi- ble and evaluable patients with a permitted accrual ranging from 18 to 25. If 3 or more out of the 25 cumulative eligible and evaluable patients at the end of stage II responded, then the study regimen would be con- sidered worthy for further investigation. Chen and Ng design gave this study an overall 90% statistical power to detect a 20% improvement in the probability of objective tumor response, an average 10% type I error rate, and an average 65% probability of early termination when the regimen was uninteresting. | No | Yes | An optimal flexible 2-stage design by Chen and Ng was chosen for this study to evaluate the null hypothesis [17]. Stage I targeted 12 eligible and evaluable patients with an accrual range from 8 and 15; if 1 or more out of the 12 patients had objective tumor response, the study would advance to the second stage. Stage II targeted an overall 22 eligi- ble and evaluable patients with a permitted accrual ranging from 18 to 25. If 3 or more out of the 25 cumulative eligible and evaluable patients at the end of stage II responded, then the study regimen would be con- sidered worthy for further investigation. Chen and Ng design gave this study an overall 90% statistical power to detect a 20% improvement in the probability of objective tumor response, an average 10% type I error rate, and an average 65% probability of early termination when the regimen was uninteresting. |
| (129) | persistent or recurrent endometrial cancer | No | A flexible, minimax 2-stage design (Chen and Ng, 1998) was chosen for this study to test the null hypothesis that the proportion responding is no greater than 5% against the alternative that the proportion responding is at least 25%. The design has an average expected sample size of 18.5 and a probability of early termination of 55%. These average probabilities are computed from the individual probabilities averaged over all permitted accrual combinations and assuming each combination is equally likely. Between 10 and 17 patients (target of 15) were to be enrolled onto the treatment component in the first stage of accrual. If the number responding is less than or equal to 0/(10-16) or 1/17, then the study would terminate early and the regimen declared uninteresting. Otherwise, with medical judgment indicating, the study will accrue to a second stage with a cumulative sample size between 21 and 28 (target of 25). The design described has 90% statistical power under the alternative specified. Type I error is set at 0.05 (one-sided hypothesis test). | No | No | - |
| (130) | Unresectable Malignant pleural mesothelioma (MPM) | No | If the difference in OS between study arms is statistically significant, then the study will be declared positive for the primary and key secondary end points and enrollment will be stopped; if the difference is not yet statistically significant, the final number of OS events will be reassessed. This provision ensures that the trial will provide 80% power to detect a statistically significant and clinically meaningful OS treatment effect at the time of the final OS analysis in the phase III part of the trial. | Not Applicable | Yes | If the difference in OS between study arms is statistically significant, then the study will be declared positive for the primary and key secondary end points and enrollment will be stopped; if the difference is not yet statistically significant, the final number of OS events will be reassessed. This provision ensures that the trial will provide 80% power to detect a statistically significant and clinically meaningful OS treatment effect at the time of the final OS analysis in the phase III part of the trial. |
| (131) | neonatal respiratory distress syndrome  (protocol) | Not reported | safety, futility or noninferiority | Not Applicable | Yes | Considering that 20% of the pregnant women receiving the full dose regimen would have a neonate with severe RDS, 1571 patients in each treatment group are required to show that the half dose regimen is not inferior to the full dose, that is the difference in severe RDS rate do not exceed 4% (corresponding to a Relative Risk of 20%), with a 1-sided 2.5% type-1 error and a 80% power. Interim analyses will be done after every 300 neonates who reach the primary outcome based on intention-to-treat, using a group-sequential non-inferiority design. |
| (132) | Cancer: relapsed solid malignancies (castration-resistant prostate cancer (CRPC) or relapsed urothelial or non-small-cell lung cancer (NSCLC) | Not reported | safety | Yes | Yes | The target toxicity rate used in this trial was 30% |
| (29) | Cancer: advanced solid tumor | Not reported | A sample size of 45 patients (with early stopping rules) was estimated to provide an accurate estimate of the MTD and to detect unexpected toxicities occurring at a 5% rate with a probability of 0.90 and at a 10% rate with a probability of 0.99. | No | No | - |
| (133) | multiple sclerosis (relapsing-remitting) | Not reported | futility RULE: all doses had less than a 20% chance of achieving a 35% reduction in CUALs compared with placebo), | No | Yes | We prospectively planned an interim analysis to be done when patients in cohort 1 had completed 3 months of study to decide whether to stop the study owing to futility (met if all doses had less than a 20% chance of achieving a 35% reduction in CUALs compared with placebo), to decide whether the sample size was appropriate, and to select two additional siponimod doses to be investigated in cohort 2, thereby facilitating optimum characterisation of the dose-response association (appendix). |
| (30) | Type 2 diabetes | No | If there was strong evidence that no optimal dose existed (i.e. no therapeutic window), the algorithm would determine the study should be stopped because of 'futility'. | No | Yes | If there was strong evidence that no optimal dose existed (i.e. no therapeutic window), the algorithm would determine the study should be stopped because of 'futility'. |
| (134) | Cencer: Rectal cancer | No |  | Not Applicable | Yes | The trial is designed to be stopped earlier if evidence of superiority of one arm over the other, futility, or unacceptable toxicity in arm A is identified. |
| (135) | Kidney Failure, End-Stage Renal Disease | No | Noninferiority and futility were analyzed after 80 patients by using the one-tailed Fisher exact test and a conditional power analysis of primary event rate trends, respectively, with early termination of the trial to be considered if conditional power to achieve a significant difference with continued enrollment fell below 15%. | No | Yes | "Early termination of the trial to be considered if conditional power to achieve a significant difference with continued enrollment fell below 15%" |
| (136) | Cancer | Not reported | - | No | No | - |
| (31) | Advanced Solid Tumors | No | - | No | No | - |
| (137) | Patients undergoing on-pump cardiac surgery for coronary artery bypass grafting (CABG) with or without concomitant valve surgery | No | O'Brien-Fleming | Not applicable | No | - |
| (138) | Advanced LKB1-inactivated Lung Adenocarcinoma | No | (1) 85% 1-tail lower confidence limit for median PFS>9.76 months; (2) <12 patients in the first evaluable 41 with at least 1 adverse event leading to treatment discontinuation. Based on the above criteria, if none of the 2 treatment arms satisfies both criteria at the same time, the experimental treatments will not be investigated further. | Not applicable | Yes | The following screening criteria will be applied: (1) 85% 1-tail lower confidence limit for median PFS>9.76 months; (2) <12 patients in the first evaluable 41 with at least 1 adverse event leading to treatment discontinuation. Based on the above criteria, if 1 treatment arm satisfy both criteria, it will be winner. if both treatment arms satisfy these screening criteria, the following sequential selection rules will be applied to identify the winner arm: (1) difference >= 5 in the number of patients without at least 1 adverse event leading to treatment discontinuation in favor of the winner arm; (2) difference >= 4 in the number of patients alive and free from progression after 1 year in favor of the winner arm. |
| (139) | Chronic Prostatitis/Chronic Pelvic Pain Syndrome | No | futility: difference was less than 20%.  the study would be stopped for success if the probability of CSD was at least 90%. | Yes | Yes | After 50% of the patients were randomized, the study would be stopped for success if the probability of CSD was at least 90%.  futility difference was less than 20% |
| (140) | Type 2 diabetes mellitus and acute coronary syndrome | No | In contrast, if the upper bounds of the 1-sided repeated CIs for the HR are ≥1.8 at all 4 unblinded interim analyses  O'Brien-Fleming-type spending function | No | Yes | If the upper bound of the 1-sided repeated CI is b1.3 at one of these analyses, the study will be considered to have met the noninferiority end point for alogliptin versus placebo  In contrast, if the upper bounds of the 1-sided repeated CIs for the HR are ≥1.8 at all 4 unblinded interim analyses |
| (141) | HIV | No | predefined stopping rules were detection of resistance-associated mutations in the integrase gene in more than two patients during the study and failure of monotherapy in more than 20 patients at any time during the study. | No | No | - |
| (142) | HIV | Not reported | Treatment failure was defined as follows: having either a decline in the primary outcomes of weight loss (>5% body mass) or CD4 T-lymphocyte (>20 cells/μL associated with percentage CD4 decline); having a score decrease of 20 points on any subscale score from the secondary outcome measure of the Medical Outcomes Study HIV Health Survey (MOS-HIV); or duration of infection more than 10 days of active infections during the 6 month trial (acquired on-study infections | No | Yes | Treatment failure was defined as follows: having either a decline in the primary outcomes of weight loss (>5% body mass) or CD4 T-lymphocyte (>20 cells/μL associated with percentage CD4 decline); having a score decrease of 20 points on any subscale score from the secondary outcome measure of the Medical Outcomes Study HIV Health Survey (MOS-HIV); or duration of infection more than 10 days of active infections during the 6 month trial (acquired on-study infections |
| (143) | HER2-positive metastatic breast cancer | No | Safety criteria: If the MTD (defined as the dose at which the estimated probability of DLT was closest to 25%) was determined before the planned number of subjects was reached, enrollment was to be stopped | No | No | - |
| (144) | Metastatic triple-negative breast cancer | No | When approximately 100 PFS events are observed in the phase III portion of the study, the DMC may recommend stopping the study early for futility if the conditional power is ≤5 % | Not applicable | Yes | When approximately 100 PFS events are observed in the phase III portion of the study, the DMC may recommend stopping the study early for futility if the conditional power is ≤5 % |
| (145) | Hypertriglyceridemia-induced acute pancreatitis | No | The corrected level of significance (p-value) will be 0.0294. At interim analysis, The following rules will be applied: 1) If any of the groups are significantly (p < 0.0294) less effective than the others and it is already visible that there is no hope for ascertaining a significant difference between the other two groups, the study will be stopped30 if. any of the groups are significantly (p < 0.0294) more effective than the others, the study will be stopped. | Not applicable | No | 1) If any of the groups are significantly (p < 0.0294) less effective than the others and it is already visible that there is no hope for ascertaining a significant difference between the other two groups, the study will be stopped. 2) If any of the groups are significantly (p < 0.0294) less effective than the others and it is already visible that there is hope of ascertaining a significant difference between the other two groups, the inferior treatment will be dropped, and the study will be continued with the rest of the two arms only. 3) If any of the groups are significantly (p < 0.0294) more effective than the others, the study will be stopped. |
| (146) | Breast cancer | No | In the first stage, 42 ER- and 28 ER+ evaluable patients would be enrolled in each arm of the respective trials. If there were fewer than 23 pCRs in an ER- trial arm or fewer than 4 pCRs in an ER+ trial arm, accrual to the respective arm would be terminated. | Yes | Yes | To allow for the early termination of ineffective treatment arms, a two-stage design was used. In the first stage, 42 ER- and 28 ER+ evaluable patients would be enrolled in each arm of the respective trials. If there were fewer than 23 pCRs in an ER- trial arm or fewer than 4 pCRs in an ER+ trial arm, accrual to the respective arm would be terminated. "Since the early stopping hurdle of 23 pCRs was only passed in the AT to CMF arm, the ER- trial was closed after the first stage" |
| (147) | Acute Myeloid Leukemia | No | Detect an EFS hazard ratio of 0.625 between the 2 arms at a 1- sided significance level of .1. "However, this trial was stopped early after the enrollment of 182 patients when it was determined that the difference between the 2 treatment arms was small." | Unclear | Unclear | - |
| (33) | Multiple myeloma | No | At the interim analysis, if it has been firmly established that the conversion rate (conversion of partial and minimal response patients to complete or very good partial response) is < 15%, the randomization will be stopped | No | No | - |

**Additional File 3:** Summary of the logistical challenges reported in the included trials

| **Record number** | **Article name** | **Logistical Challenge** |
| --- | --- | --- |
| (148) | Creative solutions to extraordinary challenges in clinical trials: methodology of a phase III trial of azithromycin and chloroquine fixed-dose combination in pregnant women in Africa | The suboptimal regional & clinical research infrastructure, inexperience in conducting clinical trials as per the International Conference on Harmonisation-Good Clinical Practice (ICH-GCP) guidelines and stringent regulatory standards of western agencies |
| (149) | An adaptive randomized trial of an intermittent dosing schedule of aerosolized ribavirin in patients with cancer and respiratory syncytial virus infection | Premature close of the trial due to dose limiting side effects |
| (58) | A phase II evaluation of aflibercept in the treatment of recurrent or persistent endometrial cancer: a Gynecologic Oncology Group study | Severe side effects |
| (150) | Lenalidomide plus cyclophosphamide, doxorubicin, vincristine, prednisone and rituximab is safe and effective in untreated, elderly patients with diffuse large B-cell lymphoma: a phase I study by the Fondazione Italiana Linfomi | The MTD (dose at which a DLT occurred in 33% of patients which is grade 3 or higher non-hematologic toxicity, or toxicity resulting in a delay of over 15 days of a planned cycle date observed during the first two courses). After a case of grade 3 motor neurotoxicity in the third course of LR-CHOP21, a protocol amendment in March 2008 called for assessment of DLT during the first three courses |
| (76) | Preoperative radiation therapy with concurrent capecitabine, bevacizumab, and erlotinib for rectal cancer: a phase 1 trial | withdrawal of financial support |
| (39) | Primaquine to reduce transmission of Plasmodium falciparum malaria in Mali: a single-blind, dose-ranging, adaptive randomized phase 2 trial | Pre-treatment infection rates in the mosquitoes were unacceptably low. |
| (80) | The ring vaccination trial: a novel cluster randomised controlled trial design to evaluate vaccine efficacy and effectiveness during outbreaks, with special reference to Ebola | community resistance, difficulty reaching remote field sites, and vaccine transportation at −80°C |
| (93) | Phase IIB/III trial of tenecteplase in acute ischemic stroke: results of a prematurely terminated randomized clinical trial | slow enrollment |
| (151) | Initial Experience with Tositumomab and I-131-Labeled Tositumomab for Treatment of Relapsed/Refractory Hodgkin Lymphoma. | slow enrollment |
| (152) | Combination treatment with varenicline and bupropion in an adaptive smoking cessation paradigm | Funding issues |
| (100) | Pazopanib in patients with von Hippel-Lindau disease: a single-arm, single-centre, phase 2 trial | Patients to come from a distance, cost of travel, and a decrement in quality of life (subjective determination by the patient) |
| (153) | A first-in-human phase I, dose-escalation, multicentre study of HSP990 administered orally in adult patients with advanced solid malignancies | Difficulty in identification of potential therapeutic targets and exploitable therapeutic index, lack of predictive biomarker and occurrence of severe toxicities |
| (154) | A double-blind, randomized controlled trial to  compare the effect of biannual peripheralmagnetic resonance imaging, radiography and  standard of care disease progression monitoring on pharmacotherapeutic escalation in rheumatoid  and undifferentiated inflammatory arthritis: study protocol for a randomized controlled trial | predicting erosive progression |
| (143) | Dolutegravir as maintenance monotherapy for HIV (DOMONO): a phase 2, randomized non-inferiority trial | mutations associated with resistance detected in the integrase gene. According to a predefined stopping rule, detection of these mutations led to premature study discontinuation. |
| (151) | Initial Experience with Tositumomab and I-131-Labeled Tositumomab for Treatment of Relapsed/Refractory Hodgkin Lymphoma | Patients’ enrollment was limited because the company stopped tositumomab production |
| (51) | Clinical Experience With Desvenlafaxine in Treatment of Patients With Fibromyalgia Syndrome (study 2) | business reason |

## **Additional File 4:** Summary of amendments (total number of studies that submitted amendments) submitted to the research ethics boards

| **Record ID** | **Amendments submitted to the research ethics boards?** | **Why were amendments submitted?** |
| --- | --- | --- |
| (35) | Yes | On 27 December 2011, the protocol was amended to enroll three additional patients to better characterize the toxicity profile of idarubicin-loaded beads at the 10- and 15-mg dose levels |
|  | Yes | Protocol Amendment (which allowed for the dosing of four  additional subjects with placebo) were drafted to provide  additional control subjects to better ascertain a relationship between the occurrence of NSVT- Non-sustained ventricular tachycardia- and JDTic administration. |
| (150) | Yes | assessment of severe DLT during the intervention |
| (155) | Yes | Phase 1 was insufficient to identify the MED (minimum effective dose), because the highest dose level tested (0.4 mg∙kg-1∙h-1) had an estimation of successful analgesia probability of 82.1%, less than the target analgesia probability of 95%, so we made an amendment to the protocol and enrolled infants to estimate the MED by increasing the dose levels of tramadol according to our previous experience with tramadol in infants |
| (156) | Yes | **The protocol was amended to allow enrollment of 100 assessable patients after closing the inferior arm, and the remaining 34 patients received TBI (total 65 patients**). |
| (39) | Yes | The study was halted on Feb 13, 2013, after eight participants had been recruited because pre-treatment infection rates in the mosquitoes were unacceptably low. Assay optimization and protocol amendments were completed, and the study restarted enrolment on the Sept 17, 2013 |
| (40) | Yes | To convert the study from single center to multicenter |
| (41) | Yes | Interim analysis was initially planned after observation of 31 deaths. However, because of the unexpected low number of deaths from start of trial until March 2009, the protocol was amended to perform the interim analysis either after observation of a total of 31 deaths or in June 2009, depending on what condition happens first |
| (43) | Yes | The development plans for palifosfamide changed after analysis showed negative phase III data regarding the addition of palifosfamide to doxorubicin in metastatic soft tissue sarcomas in several studies.This led to an amendment that closed the study to enrollment after 188 patients of the planned 464 patients were randomly assigned. |
| (44) | Yes | To increase the immunogenicity of the drug by adding a booster dose |
| (45) | Yes | To change the method of intervention administration |
| (47) | Yes | To change the study design so that higher number of people enrolled in the study |
| (124) |  | approved to allow the enrollment of an expansion cohort of 40 participant- so to increase recruitment / participant enrollment |
| (157) | Yes | To avoid the side effect occurring due to the medication |
| (158) | Yes | The protocol was amended to reduce the starting dose to 70mg twice daily following completion of the phase I trial |
| (159) | Yes | NR |
| (133) | Yes | To add a treatment arm |
| (160) | Yes | "The protocol was amended to orally or intravenously supplement 8 mEq (1 g) of magnesium sulfate during the pre-hydration regimen and as needed if magnesium levels decreased to <1.8 mg/dL or the lower limit of normal (grade 1 hypomagnesemia). This amendment was made following the observation of grade 1 to 2 hypomagnesemia in the first seven patients during the first cycle. |
| (161) | Yes | These changes were made to add research question |
| ***(33)**** | ***Yes*** | ***Amendments to include additional arms and changing randomization ratio*** |

## Notes: 7 studies did not report the reasons for amendment.

* Pediatric studies reported amendments submitted to the research ethics boards
